# A Review on Low-Dimensional Nanomaterials: Nanofabrication, Characterization and Applications

**DOI:** 10.3390/nano13010160

**Published:** 2022-12-29

**Authors:** Paras, Kushal Yadav, Prashant Kumar, Dharmasanam Ravi Teja, Sudipto Chakraborty, Monojit Chakraborty, Soumya Sanjeeb Mohapatra, Abanti Sahoo, Mitch M. C. Chou, Chi-Te Liang, Da-Ren Hang

**Affiliations:** 1 Department of Chemical Engineering, Indian Institute of Technology, Kharagpur 721302, India; 2 Department of Materials and Optoelectronic Science, National Sun Yat-sen University, Kaohsiung 80424, Taiwan; 3 Department of Chemical Engineering, Indian Institute of Technology (Indian School of Mines), Dhanbad 826004, India; 4 Department of Civil Engineering, Indian Institute of Technology, Kharagpur 721302, India; 5 Department of Chemical Engineering, National Institute of Technology, Rourkela 769008, India; 6 Center of Crystal Research, National Sun Yat-sen University, Kaohsiung 80424, Taiwan; 7 Department of Physics, National Taiwan University, Taipei 10617, Taiwan; 8 Center for Quantum Science and Engineering, National Taiwan University, Taipei 10617, Taiwan; 9 Taiwan Consortium of Emergent Crystalline Materials, National Taiwan University, Taipei 10617, Taiwan

**Keywords:** nanoparticles, quantum dots, nanorods, layered materials, nanofabrication, characterization

## Abstract

The development of modern cutting-edge technology relies heavily on the huge success and advancement of nanotechnology, in which nanomaterials and nanostructures provide the indispensable material cornerstone. Owing to their nanoscale dimensions with possible quantum limit, nanomaterials and nanostructures possess a high surface-to-volume ratio, rich surface/interface effects, and distinct physical and chemical properties compared with their bulk counterparts, leading to the remarkably expanded horizons of their applications. Depending on their degree of spatial quantization, low-dimensional nanomaterials are generally categorized into nanoparticles (0D); nanorods, nanowires, and nanobelts (1D); and atomically thin layered materials (2D). This review article provides a comprehensive guide to low-dimensional nanomaterials and nanostructures. It begins with the classification of nanomaterials, followed by an inclusive account of nanofabrication and characterization. Both top-down and bottom-up fabrication approaches are discussed in detail. Next, various significant applications of low-dimensional nanomaterials are discussed, such as photonics, sensors, catalysis, energy storage, diverse coatings, and various bioapplications. This article would serve as a quick and facile guide for scientists and engineers working in the field of nanotechnology and nanomaterials.

## 1. Introduction

Nanomaterial synthesis for numerous applications has gained interest among researchers in the last two decades. Major strides have been made in the area of nanomaterial fabrication in order to enhance the susceptibility of the nanostructured or nanomaterial-based devices. The term “nano” means dwarf and nanotechnology can be understood as the multidisciplinary science of developing, characterizing, and synthesizing materials by controlling their sizes and shapes at the scale of nanometers [1]. This class of material has the characteristic that at least one of the dimensions lie between 1 to 100 nm [2]. Such materials are noted for high surface-to-volume ratios, which enhances the properties of these materials substantially when compared to those of bulk materials. Based on the degree of spatial confinement, nanomaterials can be subdivided into four major types [3], i.e., (i) zero-dimensional nanomaterials (all the dimensions are in nanometer scale, e.g., nanoparticles), (ii) one-dimensional nanomaterials (any one of the three dimensions is of nanometer scale e.g., nanorods, nanowires etc.), (iii) two-dimensional nanomaterials (any two of the three dimensions are of nanometer scale e.g., nanosheets, nanoplates, and nano-coatings) and (iv) three-dimension nanomaterials (three dimensions are larger than 100 nm and electrons are not confined in any direction). Some examples include nanoflowers, nanocubes, nanocages, nanowire bundles, as well as other self-assemblies of lower-dimensional nanomaterials. 

The art of nanotechnology facilitates the fabrication of numerous nano and microstructures on the substrate surface. The created nanostructure arrays on the substrate surface can serve for various applications. For example, Haghanifar et al., fabricated a nano array similar to enoki mushroom structures on a polyethylene terephthalate (PET) surface using CF_4_ and O_2_ as etchants, and then SiO_2_ was deposited on the surface using plasma enhanced chemical vapor deposition (PECVD), which imparts surface superomniphobic characteristics [4]. David et al. prepared fiber Bragg grating sensor for two-phase flow in microchannels using soft photolithography and chemical etching techniques over the polydimethylsiloxane (PDMS) substrate material [5]. Qi et al. in their research demonstrate a simple approach to create hierarchical pyramidal structures over the silicon wafer using KOH and silver catalytic etching in order to achieve a superhydrophobic and anti-reflective surface [6]. Taking inspiration from hierarchical nano/microstructure from morpho butterflies scales, Gao et al. fabricated complex hierarchical nanostructures on the surface of silicon wafers using photolithography, creating a surface with elevation in heat flux and anti-reflective properties towards polarized ultraviolet, visible, and near-infrared wavelengths [7]. The fabrication of a uniform array of nanoparticles over substrate materials can be used for characterization and quantification of unknown species. For instance, Dao et al. use the process of electrodeposition in the presence of ethanol (as electrolyte) to deposit a uniform array of silver nanoparticles on the surface of silicon wafer with a precision in scale of nanometers [8]. The fabricated surface was used as substrate in surface-enhanced Raman scattering (SERS) for the detection and quantification of biological and organic compounds at very low concentrations. An interesting work by Kang et al. shows how one can create a pattern of complex micro-chambers distributed over the span of substrate using a simple but robust lithographic technique [9]. They proposed a hollow teacup-mushroom inspired microstructure whose upper surface is covered using a polymeric roof which imparts the surface with stable omniphobic characteristics without applying chemical treatments over the surface. Such micro-chambers can be loaded with the reagents, nanoparticles etc., after which these chambers will behave as micro-reactors to carry out reactions. 

## 2. Classification of Low-Dimensional Nanomaterials 

**Figure 1:**
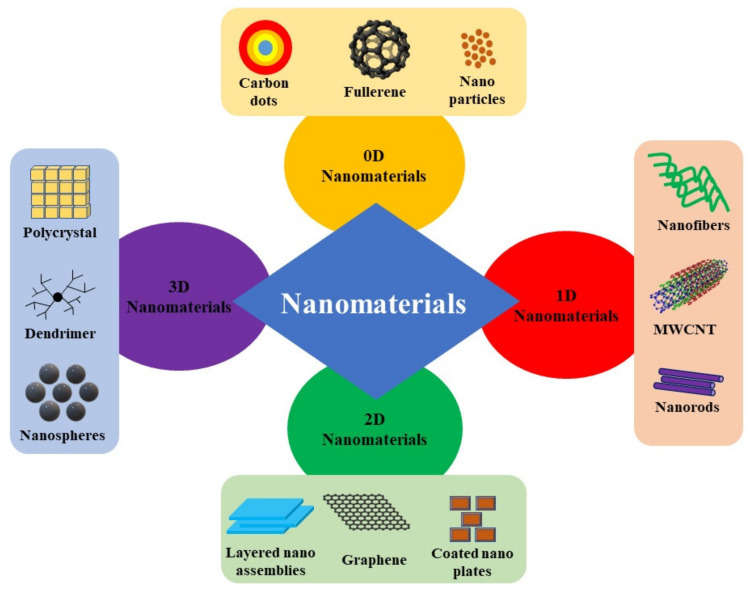
Classification of nanomaterials according to their confined dimensionality.

Breakthroughs in the field of material synthesis and fabrication techniques in the past two decades have ripened the classification of nanostructured materials (NSM). Any material is classified as nanomaterial when at least one of the dimensions of materials are in the order of nanometers. In other words, the dimensionality of a material is the major feature that discriminates against types of NSMs [10]. Generally, nanomaterials are broadly classified in four different categories, i.e., (1) zero-dimensional nanomaterials, (2) one-dimensional nanomaterials, (3) two-dimensional nanomaterials, and (4) three-dimensional nanomaterials, as shown in Figure 1. 

### 2.1. Zero-Dimensional (0D) Nanomaterials

The materials whose dimensions are all under nanometer range belong to zero-dimensional (0D) nanomaterials. Nanoparticles, quantum dots, carbon nanodots, fullerene, etc. are some popular examples of 0D nanomaterials. Significant advances have been made in the field of 0D nanomaterials during past decades. Inherent structural properties, such as the higher surface-to-volume ratio and ultra-small size, of 0D nanomaterials provides enriched active sites per unit mass [11]. Carbon quantum dots (CQDs) [12], graphene quantum dots (GQDs) [13], fullerenes [14], inorganic quantum dots (IQDs) [15], polymer dots (Pdots) [16], noble metal nanoparticles [17], etc. are some common examples of 0D nanomaterials. The reduction of dimensions imparts fabricated nanomaterials with some novel properties when compared to their corresponding bulk materials. Usually, 0D nanomaterials are either in the shape of a sphere or quasi-sphere, possessing a diameter of less than 100 nm [18].

Low capital, low inborn toxicity, and flexible surface functionalization promote the studies of carbon-based nanomaterials. Enhanced electrical and optical performance, low toxicity, and high quantum yield are some of the major advantages of 0D carbon nanomaterials [19,20]. Enhanced chemiluminescence, fluorescence, and electrochemiluminescence properties of carbon-based quantum dots promotes the use of CQDs in the fields of bioimaging and targeted drug delivery [21]. 

### 2.2. One-Dimensional (1D) Nanomaterials

One-dimensional (1D) nanomaterials possess a high length-to-diameter ratio, which could modulate their electrical, mechanical, chemical, and magnetic properties. Moreover, 1D nanomaterials also often offer high crystallinity, good uniformity and dispersion, and easy synthetic access, which surpass their bulk counterparts [22]. Particularly, one-dimensional material possesses large surface area, adaption to volume changes, pores and hollow structures, which make it suitable for hydrogen storage applications [23]. For instance, single-wall carbon nanotubes consist of sp^2^ hybridized atoms having porous nanostructures. Researchers have paid much attention towards the field of carbon-based 1D nanomaterials (nanotubes, rods, and wires) for hydrogen storage due to their increased specific surface area and reduced mass density. 

Researchers reported that nanotubes made out of inorganic materials are promising for reversible hydrogen storage as pure carbon nanotubes possess low hydrogen sorption capacity [24]. In the case of nanotubes, hydrogen can be stored in internal and external cavities of the tubes as well as in between the spaces available in nanotubes [25]. It is possible to increase the hydrogen storage capacity of carbon nanotubes by increasing the activity of tubes, leading to increased porosity and specific surface area. 

Nanorods are structurally one-dimensional nanomaterials that provide a directed path for charge carrier transport. Besides, the bandgap could be tuned by changing the radius of nanorods with quantum size effect. Literature shows that doped nanorods can increase hydrogen storage capacity as doping improves the sorption and desorption process. Nanorods are mechanically and chemically highly stable and exhibit excellent electrochemical activity, making them suitable for application in the fields of energy storage and transfer devices [26]. 

### 2.3. Two-Dimensional (2D) Nanomaterials

The remarkable progress of 2D graphene sheets in 2004 has led to its significant role in the category of 2D nanomaterials. Two researchers Andre Geim and Konstantin Novoselov showed that a single layer of carbon atoms arranged in a sheet-like honeycomb lattice possess extraordinary electrical and thermal conductivities [27,28]. Its charge carrier dynamics follows a usual linear dependence of energy on momentum. Ballistic transport over long distances results in many new transport behaviors rarely seen before, such as quantum oscillations at higher temperatures (above 100 K). The award of the Nobel Prize for groundbreaking experiments involving 2D graphene in 2010 drastically brought the attention of the public and professionals to the fields of nanomaterials and nanotechnology [29]. 2D nanomaterials are made up of atomic-layer-thick planar structures with each plane bonded to one another by weak van der Waals forces [30]. The excellent mechanical vigor and pliability of 2D nanomaterials are due to the presence of robust in-plane covalent bond and small atomic thickness [31]. Layered materials are malleable in nature, which permits their transformation to 2D nanosheets and nanoplates. The group of 2D ultrathin nanomaterials have sheet-like features with transverse dimensions of a few hundred nanometers and bearing a thickness of a few nanometers (~5 nm) [32]. Distinct topography, enhanced surface area, as well as the anisotropic chemical and physical properties of 2D nanomaterials are noted strengths for various applications [33]. Large planar surface areas of 2D nanomaterials make them very useful when surface activity matters [34]. Recent breakthroughs in the fields of 2D nanomaterials promote their applications in the fields of electronics, optoelectronics, energy storage and conversion, sensors etc. [35]. Particularly, 1D quantum confinement of electrons in the case of 2D nanomaterials results in unique physicochemical properties. The confinement of electrons in an ultrathin region of 2D nanomaterials or nanostructures provides another way to realize a classical two-dimensional electron system commonly used in high electron mobility transistors [36]. The superior transport properties and unique features for quantum transport open a new avenue for next-generation devices in electronics and optoelectronics. 

### 2.4. Three-Dimensional (3D) Nanomaterials

Three-dimensional nanomaterials can be constructed based on the arrangement and organization of a group of 0D, 1D, or 2D constituent nanostructures. Depending on the growth conditions, diverse and complex morphologies have been reported. The structure of free-standing 3D nanomaterials could be based on the forms of rods, cubes, spheres, porous spheres, foams, hierarchical dendrites, cross-linked nanorods, etc. Nano-cubes, fullerenes, dendrimers, and nanocages are some popular examples of 3D nanomaterials [37]. The dimensions of 3D nanomaterials can be beyond nanoscale, but they are not really the same with bulk materials. Compared to the bulk counterpart with similar overall size, the 3D nanomaterials could be endowed with a quantum confinement effect originating from the constituent nanostructures. On the other hand, they can keep benefits derived from larger spatial dimensional, such as the light scattering effect, which is not applicable for nano-sized objects. Thus, 3D nanomaterials have a chance to combine the advantages of both bulk materials and nanomaterials. Self-assembly from precursors is a vital principle for the preparation of 3D nanostructures. In this respect, wet-chemical synthesis is usually the most facile method for the fabrication of 3D nanostructures. The use of surfactants and templates in wet-chemical synthesis can produce many complex 3D configurations with hierarchically ordered subunits. Due to their distinctive morphologies, many 3D nanomaterials are of huge potential for applications. For examples, mesoporous structures and hollow microspheres with controlled constituent nanostructures are promising for energy storage and conversion. 3D nanostructures with hollow shells, such as mesoporous microspheres, can act as a carrier media for other nanopowder or nanomaterial. The ability to accommodate large amounts of drug make them suitable for therapeutic applications [38]. 

## 3. Fabrication Techniques

**Figure 2:**
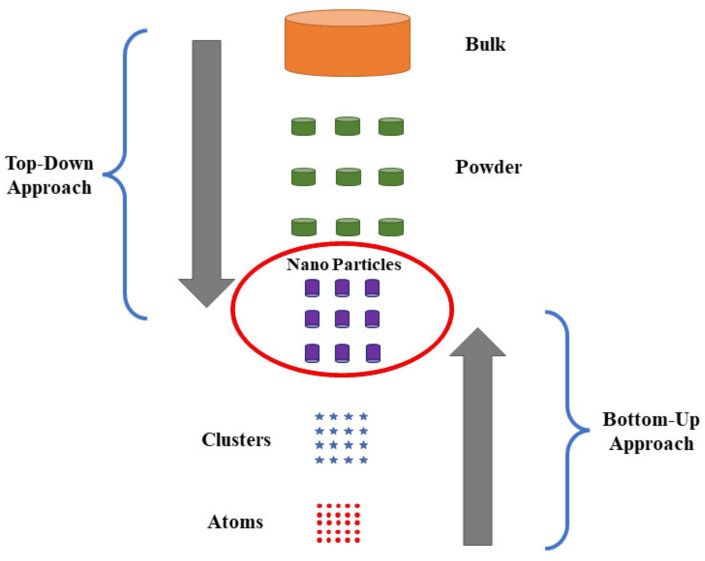
Illustration of “Top-down” and “Bottom-up” techniques for nanofabrication.

A typical methodology towards nanostructured material fabrication can be classified into two broad categories, i.e., (i) the top-down approach and (ii) bottom-up approach, as shown in Figure 2. In the top-down approach, one starts with bulk material (in orders of microscale), then breaks this bulk material using numerous techniques to form a nanoscale particle/structure, whereas in the case of bottom-up approach one starts with smallest scale possible then makes its way up the dimensions till nanoscale particle/structures are achieved [39]. Both of the above-mentioned approaches carry some merits and demerits, as summarized in Table 1 and Table 2. This section highlights their possible advantages and shortcomings and manifold fabrication techniques that come under these approaches. 

### 3.1. Top-Down Approach

Top-down approach basically revolves around removing a part of material from the starting bulk solid to achieve fabrication in orders of nano/micro scale [40,41]. In layman’s language, we can think of the top-down fabrication approach as a process similar to that of a sculpture artist’s process of sculpting a sculpture from a block of stone. The top-down approach is comparatively simpler as the process involved depends either on the removal or break down of bulk material or on miniaturizing the existing bulk fabrication protocol to produce the desired micro/nano structure with appropriate size, chemical, and physical properties. Many methods in this approach are well-suited to current industry technology. Fabrication over a large substrate is possible and reliable. Yet, it could come with the price of imperfections induced on surface structures. Traditional top-down techniques used for creating nanostructures and nanopatterns are discussed as following. 

#### 3.1.1. Lithography

The term lithography is derived by joining two Greek letters, i.e., lithos means ‘stone’ and graphia means ‘to write’ [42]. Lithography was first invented by Alois Senefelder, a German playwright and actor in 1796 in the United Kingdom. However, the process of lithography gained popularity during the mid-1900s as it inspired researchers towards the discovery of practical and quicker printers. The process of lithography itself can be broadly subdivided into two categories, i.e., (i) masked and (ii) mask-less [2]. In masked type lithography approaches, the nano/micro patterns are transferred on the surface using a template or mask. Photolithography [43], contact lithography [44], X-ray lithography [45], nanoimprint lithography [46], soft lithography [47] etc. are some popular examples of masked lithography. On the contrary, in the mask-less lithography process, nano/microstructures are transferred on the surface without using a mask. Scanning probe lithography [48], focused ion beam lithography [49], electron beam lithography [50], etc. are some popular examples of maskless lithography. 

##### Photolithography

**Figure 3:**
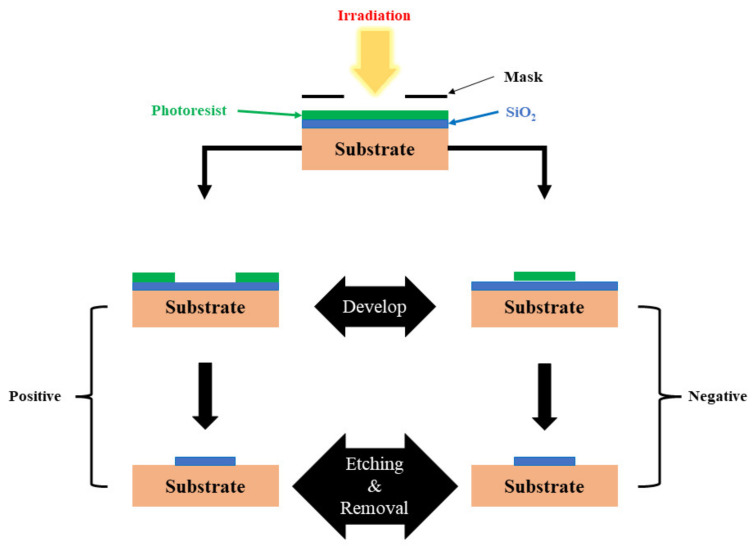
Types of photoresists.

Photolithography has been a practical and widely used technique used for the fabrication of micro/nano structures over various surfaces [51,52]. The process as explained earlier is a mask-based lithography technique where the final pattern to be transferred on the surface depends on the type of photoresist being used. This technique is most commonly used in the sector of semiconductors as such a process is easily scalable. A suitable light-sensitive photoresist is initially deposited on the surface, which is then covered with a lithographic mask. The result of this photolithographic process relies on the structure image presented on the mask. As shown in Figure 3, a photoresist used on the surface can either be a positive photoresist, in which the surface exposed to the light irradiation undergoes photochemical degradation and gets etched away, leaving solid nanostructures behind, or can be a negative one, in which the photoresist is directly deposited on the substrate surface. After light irradiation, the exposed portion of the photoresist becomes insoluble to the photoresist developer. In contrast, the covered portion of the photoresist can be dissolved by the photoresist developer [53]. Photolithography has demonstrated significant promise in patterning solution-processed nanomaterials for the fabrication of light-emitting devices. For instance, a direct in-situ photolithography method for patterning perovskite quantum dots (PQDs) on lead bromide complexes was recently reported by Zhang et al. [54]. Their work demonstrated the manufacture of efficient red, green, and blue light-emitting PQDs, which were patterned with a minimum feature size of 5 μm and a thickness of 10 μm. The obtained PQD pixels showed good absorption of blue and UV light for use in color conversion.

##### Contact Lithography

Contact lithography is also known as proximity lithography, which is a similar approach to that of photolithography, but with a difference that the photomask in this case is in close proximity to that of photoresist layer over the substrate which allows the light passing to form a 1:1 structure over the photoresist [55]. The primary merit of contact lithography is that the system is free of complex projection optics between object and image. Resolution is one of the factors to be considered. When the light propagates through the photoresist, diffraction cause the image to lose contrast with increasing depth into the photoresist. It can be mitigated by using a thinner photoresist to improve image contrast [56]. Recently, Pugachev et al. demonstrated the feasibility of manufacturing low-cost micro masks using contact lithographic methods, allowing for the straightforward scaling up of 2D materials microstructure fabrication [57]. The constructed micro mask can achieve resolutions on the order of micrometers (~0.6 μm). 

##### X-Ray Lithography

X-ray lithography is a slightly advanced version of conventional photolithographic technique which uses X-ray beam as the source of irradiation instead of light source. The major advantage of X-ray lithography is that it can operate in shorter wavelengths and can provide a high-resolution reserve for Moore’s law requirement that conventional lithography techniques failed to meet [58]. Moreover, the use of X-rays instead of conventional light sources provides deeper penetration into the resist layer, which ultimately provides higher resolution and better aspect ratio [59]. Because of the high precision and accuracy of X-ray lithography, this process is used for the fabrication of micromixers [60]. With the support of precise X-ray lithography using a synchrotron radiation source, Gentselev et al. succeeded in the fabrication of high-quality planar structures for use in terahertz optics [61]. It was shown that far greater control over cell size, wall width, and thickness can be achieved, compared with previous lithographic technology skills. The obtained self-supporting perforated X-ray masks can be used to fine-tune the parameters of beams of electromagnetic radiation in the terahertz and subterahertz ranges, and these masks feature high contrast X-rays (λ = 0.6–14 Å), allowing them to be used in conjunction with both positive and negative X-ray resists.

##### Nanoimprint Lithography

Nanoimprint lithography is one of the most attractive lithographic techniques. The beauty of nanoimprint lithography lies in its capability of producing periodic arrays of nanostructures over large areas with high throughput and high aspect ratio [46,62]. The process uses a well-defined lithographic stamp which imprints a moldable polymeric resist into an array of well-defined nanostructures. The use of this lithographic stamp makes this process commercially available and reusable, ultimately making the whole process cost efficient and technologically straightforward in terms of application. Nanoimprint lithography requires only repetitive simple nano-stamps for the fabrication process to take place. This makes the process much simpler with regard to the fabrication of different types of hybrid nanopatterns. Many researchers used nanoimprint lithography for achieving various nanostructures over the surface. For instance, Choi et al. adopted nanoimprint lithography to create polymeric nanopores to measure transient currents generated by the translocation of DNA molecules [63]. 

##### Soft Lithography

Soft lithography is a technique which involves the transfer of structures over the substrate using soft elastomeric masters (e.g., stamps, molds, photomasks etc.) [64]. Compared to other fabrication techniques, the soft lithography process includes benefits in terms of cost, setup, and high throughput without a compromise in terms of resolution which can vary from nanometer to micrometer precision [65,66]. As fabrication using soft lithography is a physical contact-based micro/nano fabrication technique, we can say that the resolution of the technique is limited by the van der Waals radius. Moreover, the elastomeric properties of the nanopattern stamp imparts the process with the ability to conform non-planar shapes of the substrates with the inbuilt effect of inducing long range distortions to the nano/micro patterns/structures transferred over the substrate, and the significance of these distortions varies from application to application [67]. 

Soft lithography can be used in various applications, such as biosensors, directional cell affinity, bio-sensing, cell sorting, tissue engineering etc., which make this lithographic technique suitable for applications in the field of medical sciences [68]. Recently, Gan et al. reported a cost-effective, patterned growth of high-quality 2D transition metal dichalcogenides using the soft lithography technique [69]. A liquid precursor (Na_2_MoO_4_) was patterned initially on the growth substrate. Then, CVD was employed to convert the precursor patterns to monolayer, a few layers, or bulk TMDs, according to the precursor concentration. They showed that the photodetectors made of MoS_2_ line patterns achieved an excellent responsivity of 7674 A W^−1^ and a good external quantum efficiency of 1.49 × 10^6^%. 

##### Scanning Probe Lithography

**Figure 4:**
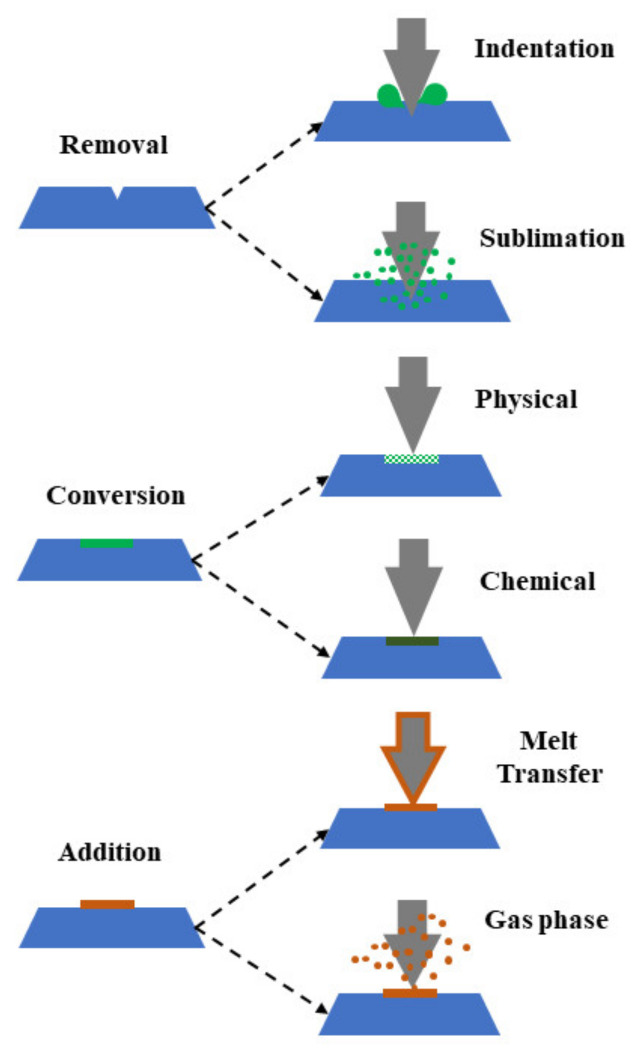
Schematic diagram of scanning probe lithography.

Scanning probe lithography, also known as direct-write nanolithography, is a state of art lithographic technique, as shown in Figure 4. In scanning probe lithography, nano/micro patterns are created using a nanometer sharp tip over the sample substrate by inducing either mechanical, electrical, diffusive, or thermal effects [70]. In the operation, thermal energy from a nanometer thick probe can be transferred to the local substrate to transfer the nano/micropatterns [48]. With this technique, 3D nanostructures are created by mechanically moving a tiny or nanoscale stylus over a surface to create patterns at the nanometer scale. With the use of the stylus, 3D structures may be created by adding material to the substrate’s surface or by scraping away portions of the surface. Dip pen nanolithography, which involves transferring a substance from a stylus to the substrate, is mostly used to chemically functionalized surfaces [71]. Contrarily, intricate 3D patterns in polymer resists have frequently been created by directly patterning a surface by scraping away portions of it with a stylus.

##### Focused Ion Beam Lithography

**Figure 5:**
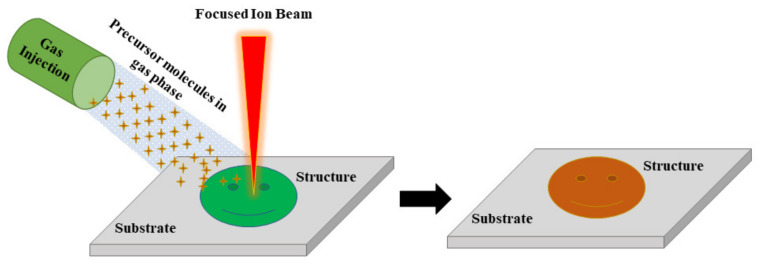
Focused ion beam lithography.

**Figure 6:**
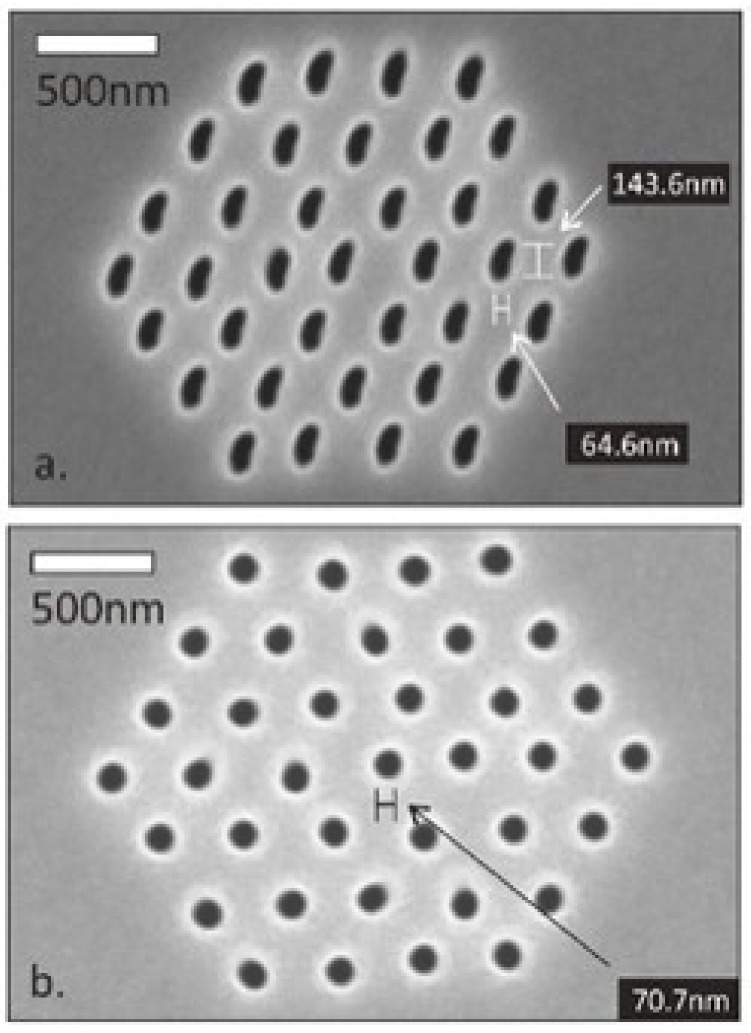
Nanopore arrays fabricated by Ga^+^ ion milling in bare (**a**) and metallised (**b**) silicon-nitride membranes. The distorted pore geometries are result of the beam defocusing effect of electrically charged dielectric membrane [76].

Focused ion beam lithography, popularly known as focused ion beam milling, is now arising as one of the most popular top-down approaches for nanofabrication [72,73]. It works based on the principle that ions have higher atomic weight when compared to electrons, and a focused beam of ions can remove material much faster compared to electrons due to its higher linear momentum. The major advantages of focused ion beam lithography are that it doesn’t require any specialized mask for its operation and can produce high-resolution nanostructures. As shown in Figure 5, during the operation, a focused beam of ions is combined along with precursor molecules that etch and deposit the precursor material locally [74]. The precursor molecules typically in gas phase are transported to the substrate’s surface by a gas injection system while a focused beam of ions scans the substrate surface forcing the growth of precursor material over the surface in a shape as directed by the focused beam of ions. This lithographic technique can attain a resolution as high as a few nanometers. In the past, researchers showed evidence of achieving a resolution of 3-nm for platinum-based nanostructure growth [75]. Fürjes et al. adopted the FIB milling process for the preparation of solid state nanopore arrays for molecule sensing [76]. They found the pore diameter depends on a few factors, such as the material composition, the thickness of the membrane, the applied milling time, ion dose, current, and energy. Besides, the reproducibility of the FIB nanoprocessing can be improved by neutralization (electrostatic grounding) of the sample surface, applying an additional conducting layer. The suppression of the electrical charging of the dielectric layers would preserve the ion beam shape, as shown in Figure 6. It can be seen that the distortion of the pore diameter could be reduced significantly, and the pore size variations kept reproducibly below 5 nm. 

##### Electron Beam Lithography

Electron beam lithography is a direct lithographic process that utilizes the power of focused beams of electrons to transfer an array of nano/micropatterns over the surface of substrate without the need of any mask. The process of electron beam lithography can either be additive (i.e., material depositing) or subtractive (i.e., removal of material) in nature [77]. This type of lithography process is also used for preparing stamps used in nanoimprint lithography. During the operation, the desired nano/micro pattern is directly scanned over the resist surface by using an energetic electrons beam. The use of electrons provides an excellent depth of focus, which is corrected up to several hundred microns of height. A major drawback of electron beam lithography is that the process is cost inefficient, and therefore not practical for large scale fabrication processes [78]. Recently, Tu et al. demonstrated a resist-free lithography of metal–organic frameworks (MOFs) at the micro- and nanoscale by X-ray and electron-beam lithography [79]. Conventional MOF patterning methods suffer from low resolution and poorly defined pattern edges. The benefit of this method is that it preserves the porosity and crystallinity of the patterned MOFs while avoiding etching damage and the contamination of fabricated nanopatterns. The generated high-quality patterns fall in the mesopore region, having excellent sub-50 nm resolution.

#### 3.1.2. Sol Gel Method

A common and practical way for creating nanoparticles with various chemical compositions is the sol-gel method. The creation of a homogenous sol from the precursors and its transformation into a gel form the foundation of the sol-gel process. The leftover gel is then dried after the solvent in the gel is removed from the gel structure [80]. The main benefit of the sol-gel process is that it produces stable surfaces with a high surface area. The experimental settings used are connected to the chemical and physical characteristics of the materials produced by the sol-gel process [81]. The sol-gel method of fabrication initially involves preparing a uniform regular gel phase. Later, the solvent evaporation from the gel matrix is performed so as to achieve a thick multilayer coating on the surface’s substrate. There are three different ways of removing solvent, i.e., (a) evaporation, (b) supercritical drying, and (c) freeze drying, upon which the structure of coating depends. For instance, in order to achieve aerogels, the supercritical drying of gels is performed (having regular and ordered structure). Protracted evaporation of gels provides xerogels, whereas to fabricate cryogels, freeze drying of gels is preferred. This flexibility of sol-gel fabrication process allows the fabricator/manufacturer to extend their horizons of applications [82]. The advantage of using the sol-gel method lies in its ability to control morphology and particle size as these parameters in the sol-gel method depend on the reaction mechanism. 

#### 3.1.3. Template Etching

Template etching is a chemical-based etching technique where nanofabrication is done using chemical etching reagents directly on substrate with the use of template [83]. The template is prepared using spin coating or electron beam lithographic techniques. The most commonly used template materials are ordered nanoporous membranes, which contain ordered structures or pores through which the chemical etchant passes to the substrate surface. The size of nanomaterial over the surface is tuned by either changing the pore sizes or by tuning the anodic or cathodic potentials of the substrate surface. This type of template etching can produce long range arrays of structures with nanoscale features over the surface. The final feature in this type of micro/nano fabrication appears after the removal of the template.

### 3.2. Bottom-Up Approach

Contrary to the top-down approach, the bottom-up approach uses chemical and mechanical forces operating at nano/microscale to assemble the basic units into larger structures. Motivation for bottom-up approaches comes from natural biological systems, where nature utilizes various chemical forces to fabricate/produce essentially all the edifices needed by life. Researchers always try to replicate/simulate nature’s ability to produce nano/micro clusters of specific elements, which can then self-assemble as one to form more-elaborate structures. In the bottom-up fabrication approach, the controlled segregation of molecules or atoms occurs as they come together and assemble as one unit. Bottom-up synthesis can be carried out either in gas phase or in liquid phase formation. 

#### 3.2.1. Plasma Arching

Plasma is one of the fundamental states of matter which consists of electrons and molecules in ionic states. It maintains a condition of overall neutrality, although there may be a net positive or negative charge on certain particles. The plasma arcing method requires an ionized state of gas atoms, for which high energy is necessary to peel off the electron from its valence shell to obtain a positively charged atom. An electrical arrangement consisting of an anode and cathode is developed providing a sufficient amount of electric field to convert the atoms into ions. Electrodes used are usually made up of conducting materials or mixtures of conducting and non-conducting materials. The generation of contracted plasma uses inert gas as a heat source. The emission of electrons takes place from one electrode due to the presence of a high potential difference causing an electrical breakdown. A sudden avalanche of electrons results in the formation of an arc in the zone between the electrodes. Positively charged ions travel at a high velocity and are driven by the applied bias voltage toward the cathode and get deposited as nanoparticles. The depth of deposition corresponds to a few atomic layers and all particles so formed are mutually separated. The average temperature of the arc in cold plasmas is generally higher than 104 K. 

#### 3.2.2. Chemical Vapor Deposition (CVD)

**Figure 7:**
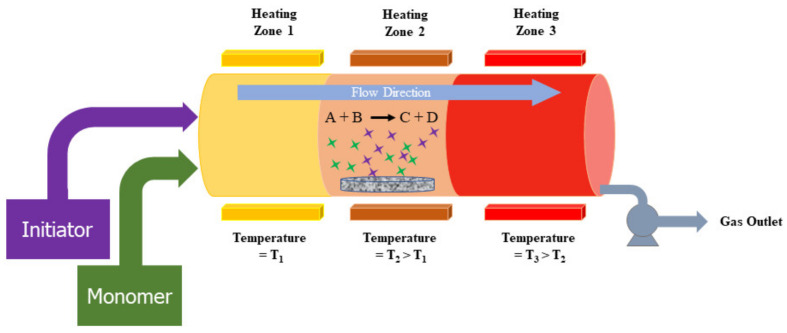
A three-zone chemical vapor deposition (CVD) system.

The CVD technique uses precursors of reactant gases in the reaction chamber. Molecules of these precursor gases pass over the substrate inside the reactor and then the deposition of molecules takes place over the substrate’s surface [84]. Figure 7 illustrates the detailed process of three-zone CVD design. The CVD technique is one of the most welcomed synthesis tools because it can prepare almost any type of inorganic material with reasonable cost. It can produce both nanomaterials and thin films. Deposition using CVD requires a high temperature. Precursor handling is sometimes a tricky process and the choice of proper substrate is very important for CVD. These factors need to be considered for the CVD process.

#### 3.2.3. Atomic Layer Deposition (ALD)

**Figure 8:**
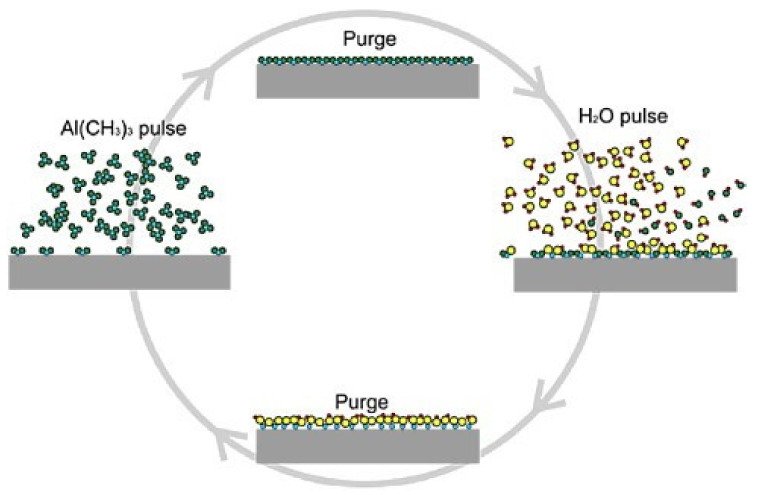
Schematics of an ALD growth cycle. Reproduced with permission from [86]. Elsevier, 2014.

Atomic layer deposition (ALD) can be regarded as a variation of chemical vapor deposition (CVD) in which gaseous reactants (precursors) are injected into the reactor system one at a time in a sequential way to generate the desired products by surface chemical interactions [85]. In order to prevent gas phase reactions, ALD pulses the precursors alternately, one at a time, and separates them with inert gas so that the reaction terminates once all the reactive sites on the surface are consumed, as shown in Figure 8 [86]. Based on alternate interactions between gaseous precursors and solid surfaces, ALD shows excellent capability in homogeneous deposition of conformal layer with tunable cross section, even on intricate three-dimensional surfaces. ALD is suitable for ultra-thin film deposition possibly down to the atomic level. Nowadays ALD is frequently employed in microelectronics fabrication for the deposition of high-κ gate oxides, ferroelectrics, metals, and nitrides [87].

#### 3.2.4. Pulsed Laser Deposition (PLD)

Pulsed laser deposition (PLD) is a form of physical vapor deposition in which a laser with a high-power density and narrow frequency bandwidth is utilised as a source to vaporize the target material to form a plasma plume. With the repetition of laser pulses, ions from plasma could condense and aggregate on a nearby substrate with controlled temperature. Then, thin films of different materials can be grown on the substrate. The operation can be carried out in UHV or in the presence of some background gas. For instance, oxygen can be used as the background gas during oxide materials deposition. It can be used to grow a high-quality oxide thin film as the film formation rate can be precisely controlled by the number of laser pulses. The working mechanism of the full PLD process is complex due to the involvement of laser ablation and plasma dynamics. In general, there exist five crucial aspects to be considered for high-quality crystalline materials grown by PLD [88]: (1) Absorption of the laser by the target material. (2) Laser ablation of the target material and the generation of the plasma. (3) Plasma physics. (4) Deposition of the ablation material on the substrate. (5) Nucleation and expansion of the thin film. It is a versatile synthesis technique for many kinds of materials, including semiconductors, superconductor, complex oxide films, and various nanostructures.

#### 3.2.5. Laser Pyrolysis

Laser pyrolysis is another bottom-up approach used for the synthesis of nanoscale systems. The present laser pyrolysis process is not as popular as the laser ablation process, but it is a very useful process when dealing with the synthesis of carbide and ceramic nanostructures [89]. In a typical laser pyrolysis process, the gaseous-phase precursors are introduced to a chamber by a carrier gas (e.g., argon) where the gaseous-phase precursors meet the laser beam. The high-power laser beam (e.g., 2400 W) generates elevated localized temperatures which trigger the nucleation and growth of nanoparticles. The nanoparticles are then collected by a catcher equipped with a filter. The laser pyrolysis is characterized as a gaseous-phase process, with additional functionality to handle liquid reactants. When liquid reactants are necessary, those reactants can be vaporized or turned into microscale droplets (e.g., via an ultra-sonication process). Thereby, they can be introduced to the reaction chamber. Since this technique mainly applies the thermal energy induced by high-power lasers, lasers (e.g., CO_2_) operating in continuous modes are often applied.

#### 3.2.6. Molecular Beam Epitaxy (MBE)

**Figure 9:**
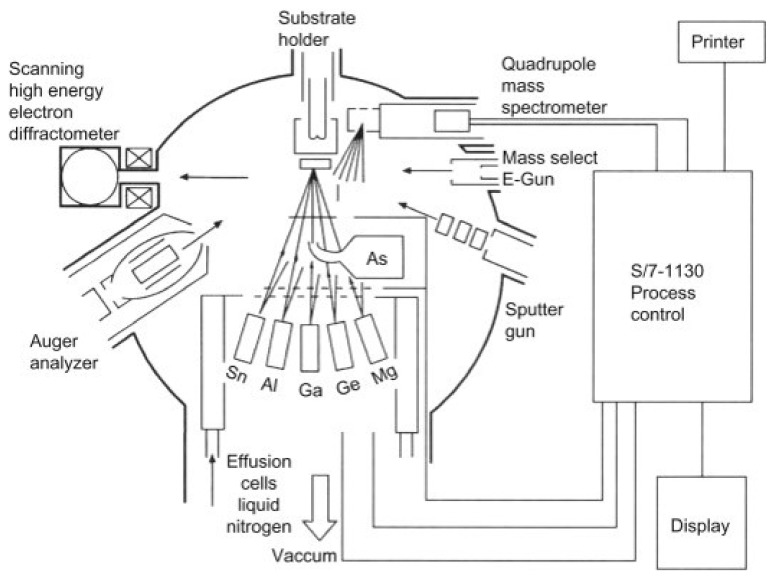
Schematic of MBE system. Reproduced with permission from [91]. Elsevier, 2012.

Molecular beam epitaxy is a physical evaporation process with no chemical reactions involved. The basic difference between MBE and other epitaxy systems is that the former does not involve any chemical reactions and is associated with a simple physical evaporation process. This method depends on the principle of vacuum evaporation where thermal molecular and atomic beams are directly impinged on a heated substrate under ultra-high vacuum conditions [90]. An MBE system is illustrated in Figure 9 [91]. The first major advantage of the MBE process is that it is a comparatively low-temperature process as compared to vapor phase epitaxy. The low-temperature characteristic of this process enables it to reduce the auto-doping effect. The second advantage of MBE is that one can have precise control over the doping process and layer thickness. One can achieve a growth rate as low as 0.01 µm per minute up to a maximum of 0.3 µm per minute, allowing for the ultra-precise control of layer growth. This is crucial for the preparation of semiconductor nanostructures and heterojunctions. With the advancement of chip technology, it is critical to reduce device feature size to atomic levels, and thus the precise control of thickness of the epitaxial layer will be important in future.

#### 3.2.7. Molecular Self-Assembly (MSA)

Self-assembly is a process in which an ordered composite structure is formed by mutual interactions between disordered building blocks through a spontaneous organization. This attraction can occur between macro, nano, or mesoscopic structures over a wider range of composition, geometries, and functionalities [92]. Nanoparticles based self-assembled layers over a surface occur due to the balance between attractive forces and repulsive forces [93]. Surfaces created using self-assembly process possess higher order of flexibility. The molecular self-assembly (MSA) process is an ensemble of sequential chemical synthesis, covalent polymerization, self-organizing synthesis, and molecular self-assembly. MSA is a process in which atoms or molecules assemble together in equilibrium conditions to form a stable and well-defined nanophase by non-covalent bonds. The molecular self-assembly process is highly capable of fabricating nanostructures in the range of 1–100 nm [94]. In order to create complex nanostructures using a self-assembly process, critical parameters, such as the well-defined geometry and the specific interactions between the basic units, are requisite significant consideration.

## 4. Characterization Techniques

**Figure 10:**
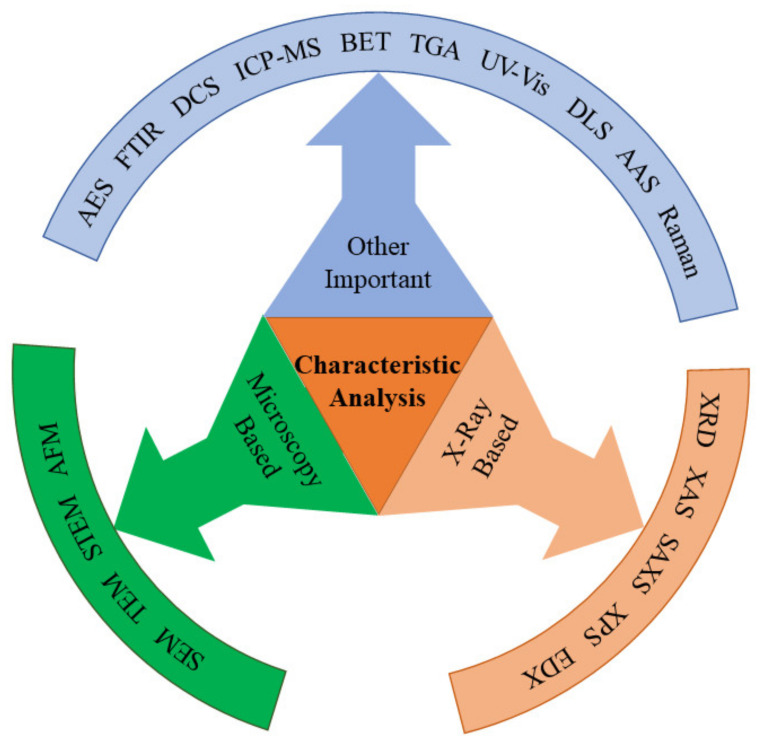
Characterization tools for nanomaterial characterization.

The experimental methods that are used to quantify the dimensions, crystal orientation, elemental composition, and other physical and chemical properties of synthesized nanomaterials are termed as characterization techniques [95]. Figure 10 depicts characterization tools with different technological basis. Essential characterization approaches for semiconductor nanomaterials are summarized in Table 3 [96,97,98,99,100,101,102,103,104,105,106,107,108,109,110,111,112,113,114,115,116,117,118]. 

### 4.1. X-ray-Based Characterization

X-ray diffraction (XRD) is one of the most commonly used techniques when it comes to structural determination for nanomaterial. XRD works on the principle of constructive interference of monochromatic X-ray and Braggs law. According to Bragg’s law, the diffraction of an X-ray beam from a crystalline surface is a characteristic of material and depends on wavelength of X-ray, scattering angle, and inter-planar distance according to Equation (1).

2*d* sin*θ* = *n*λ (1)

where *d* is lattice spacing, *θ* is incidence angle, *λ* is wavelength, *n* represents the order of constructive interference (diffraction order). The composition of nanoparticles can be determined by comparing the obtained data with available reference pattern data provided by the International Centre for Diffraction Data (ICDD). The crystallinity of the materials can be determined by the sharpness of the diffraction peak from the analyzed sample, i.e., amorphous material in general exhibits broad peaks whereas high-crystalline samples yield a very sharp peak. One can also provide information regarding a material’s lattice parameter, phase nature, and crystalline grain size using a small amount of powdered sample and the Debye–Scherrer formula given by Equation (2), where *D* is crystallite size, *k* is Scherer’s constant (*k* = 0.94), *β* represents full width at half maxima (FWHM), and θ is location of peak [119]. Velavan et al. used XRD for the analysis of silver nanoparticles [120]. They reported that the average size of the nanoparticles formed by the reduction of silver ions using Erythrina indica flower extract is ~ 28.19 nm which is obtained using the Debye–Scherrer formula.

(2) $$D=\frac{k\lambda }{\beta \cos\theta }$$

The X-ray absorption spectroscopy (XAS) technique involves transitions from ground state to the excited state by absorption of X-rays [97,121]. Each element is unique in terms of its atomic/elemental composition and binding energy. XAS measures this binding energy as the function of X-ray absorption coefficient, which is unique for every material, thus it can be considered as a fingerprint of material. XAS in general measures displacement to the excited state from the core state and the continuum. It is possible to study the local structure of a target element with XAS without interference from the proteins, water, or air molecules involved in the absorption process [97]. The merit of XAS technique lies in its ability to investigate transition metals, e.g., Au(I), Ga(III), Zn(II), etc., with full and empty d-shells [122]. Lopes et al. used in situ XAS study for the determination of activation of palladium nanoparticles [123]. 

Small-angle X-ray scattering (SAXS) is an analytical technique that uses scattering at different angles to determine how much X-ray is scattered by a sample. In this regard, SAXS probes modifications of sample’s electron density caused by X-ray scattering to generate contrast [124]. As SAXS provides inverse space data, atomic scale variations in electron density will scatter X-ray beams to high angles while nanometer scale variations will scatter them at low angles. Therefore, SAXS is a technique that is useful for examining complex structures over a large distance with a small angle of incidence. Particles constructed through sol-gel reactions, aerosols, micelles, minerals, and other particles have been studied using SAXS. Nano-porous structures can also be examined using SAXS since it measures only the difference in electron density between colloidal particles and nanoporous structures [125]. Li et al. reported that in-situ nanoparticle synthesis, nanoparticle assembly, and operando investigations of catalysts and energy storage materials can be analyzed using SAXS [124]. Garcia et al. performed an in-situ SAXS analysis for the determination of silver and bimetallic silver-gold nanoparticles produced by the wet- chemical reduction synthesis [126]. 

X-ray photoelectron spectroscopy (XPS) is an analytical method that uses photoelectrons stimulated by X-rays (usually Mg _K_ or Al _K_) to evaluate surfaces up to the depth of 10 nm [127]. During analysis, ultra-high vacuum (UHV) is required for experimental setup as any hindrance of foreign particles is not allowed while analysis. The majority of contemporary spectrometers have variable tolerance ranges of roughly 50 m–1 mm. Therefore, common sample sizes in the 5 mm × 5 mm range (even for handling considerations) are desired. Furthermore, XPS examination of particles has significance that extends beyond 100 nm and is even relevant to bigger particles. Understanding the surface characteristics of NPs as well as the composition and thickness of coatings on NPs requires the use of XPS [128]. Important details, including NP composition, impurity presence, functionalization consistency, surface adsorption capacity, and layer and coating thickness, can be extracted with a variety of methods. Since XPS can distinguish between a variety of functional groups on the surface (down to a depth of 10 nm), the information gathered is helpful for understanding potential interactions between nanocomposites and their surroundings. XPS can offer information of the surface composition and binding interactions of spontaneously generated nanocomposites made of two or three components with convenient commercial XPS instruments [129]. Sublemontier et al. used the XPS technique for the determination of isolated Si/SiO_2_ nanocrystals onto the surface without any substrate interactions and charging of silicon nanoparticles [130].

Energy dispersive X-ray (EDX) technique uses characteristic X-rays produced via interaction of sample with energetic electron beam irradiation to identify elements involved [131]. Usually, when an incoming electron strikes an atom of the sample, it knocks off an electron from the metal’s K-shell orbital (*n* = 1), leaving a vacancy or hole. X-rays are released if an electron from another shell fills in the empty space (electron transitions). K_X-rays_, L_X-rays_, and M_X-rays_ are the names given to electronic transitions to the K-shell (*n* = 1), L-shell (*n* = 2), and M-shell (*n* = 3), respectively. Each chemical element undergoes these transitions, and this is the foundation of EDS detection systems for electron microscopy [132]. EDX makes use of the X-ray spectrum produced when a solid material is exposed to a focused beam of energetic electrons. The emission of X-ray energy is directly proportional to the atomic number of the substrate’s element with which the electrons interact [133]. The accuracy and sensitivity of the instrument, the spatial resolution (determined by the penetration and spreading of the electron beam in the specimen), and the sample preparation method all affect how effectively and reliably the analysis is performed using an electron beam to obtain a localized chemical analysis [134]. Patri et al. studied the distribution of titanium dioxide nanoparticles in mice following intravenous and subcutaneous injection using energy dispersive X-ray technology [135]. 

### 4.2. Microscopy-Based Characterizations

The high-resolution scanning electron microscope (SEM) is a great tool for determining the size and form of nanostructures since image capture and sample preparation are both very rapid and easy processes. Even while the SEM picture is a two-dimensional (2D) depiction of the three-dimensional (3D) objects from a certain viewing angle, it does contain some 3D information that, when combined with model-based measurement, may be used to accurately reconstruct the shape down to the nanometer level. The secondary electrons (SEs) and/or backscatter electrons (BSEs) produced when the sample is hit by the primary electron (PE) beam provide the signal that generates a SEM picture [136]. In the top micron or so of a specimen, SEM can reveal details about the surface topography, crystalline structure, chemical composition, and electrical behavior [137]. By choosing particular types of emitted electrons, referred to as SE (energy lesser than 50 eV) and BSEs (energy greater than 50 eV), respectively, morphological/topological contrast and compositional information can be independently acquired [138]. Typically, SEM can be classified into three different types based on the manner and the environment that they work with i.e., a) conventional SEM (CSEM), which operates at high vacuum pressure (~10^−8^ torr), b) low vacuum SEM (LVSEM), which is similar to CSEM but can also operate at elevated pressures (0.2 to 2 torr) with a dehydrated environment around the sample, and c) environmental SEM (ESEM) operates at elevated pressures (0.2 to 20 torr) and allows samples to be placed at hydrated environment [139]. Zheng et al. analyzed nanoparticles present in tissue or cultured cell thin slices using SEM and X-rays [140]. 

By passing an electron beam through a thin specimen, transmission electron microscopy (TEM) makes it possible to see the sample’s inside in great detail. Due to the unique information at high resolution, TEM is frequently employed in nanomedical research and is capable of revealing the subtle interactions between nanoparticulate and their constituent parts. Because electron beam has very small wavelength (about 100,000 times shorter than visible-light photons), TEM may produce images with sub-nanometer resolution, or around 0.2 nm [141]. The electron energy is sufficient to allow the electrons to pass through the sample. Even with extremely small single crystals (from 25–500 nm) at very small amounts of material, TEM paired with precision 3D electron diffraction tomography technology can provide vital structural findings for crystal structure characterization [142]. To examine the development of electronic material in operational settings, it is possible to use an in-situ transmission electron microscope (TEM) with atomic resolution and an external field. The great spatial resolution and flexible characterization capabilities of TEM have made it a popular tool for studying the structure-property relationships of materials and devices. The new aberration correction method has increased the TEM’s spatial resolution to 0.5 Å. This makes it possible for researchers to observe the atomic structure of materials up close. TEM can offer structural and chemical information, and even valence state in addition to morphology. A real-time approach for characterizing and manipulating materials using external stimuli, including electrical, mechanical, thermal, optical fields, and liquid/gas environments, is realized by the recently developed in-situ TEM technology [143]. Franken et al. summarized the use of TEM for high-resolution 3D images in the field of soft-matter research [144]. 

In semiconductor nanomaterials science, site-specific image analysis with nanometer and sub-nanometer scale resolution are usually provided by scanning electron microscopy, transmission electron microscopy, and scanning transmission electron microscope (STEM) [109]. In STEM, a probe electron beam is focused to a tiny region of few angstroms, which is then moved by deflection coils to scan over the sample. It is possible to create either a bright-field STEM picture or a dark-field STEM image, depending on the transmitted electrons that are utilized to create the image. Deflection coils beneath the specimen act simultaneously to adjust for the produced tilt, retaining only the lateral-shift component of the movement. The beam deflected by lenses is then scanned point by point in a raster mode over the specimen surface [145]. Z-STEM, which uses atomic number contrast scanning transmission electron microscopy, has an excellent capacity for extracting chemical and structural data from individual nanostructures at the atomic scale. Z-STEM is a type of STEM that primarily employs a high-angle annular dark field (HAADF) detector to get an indirect image of the object’s structure from an incoherent image. Z-STEM is a perfect tool for studying core-shell structures at the atomic level because, unlike traditional HRTEM, which uses phase-contrast imaging to reveal the crystalline nature of the particles, the intensity seen in the Z-STEM images depends on the scattering power of the atom being imaged. This yields chemical information concurrently with structural position [146]. The accurate characterization of shell form, coverage, and chemical composition is made possible by the differential intensity between the core and shell, heterodimer, or heterotrimer shown in Z-STEM pictures. 

For micro/nanostructured coatings and thin nanostructures, atomic force microscopy (AFM) is a significant tool for surface science study. With this adaptable method, local areas may be studied in the presence of air or liquid using conventional AFM or electrochemical AFM to capture high-resolution nanoscale pictures [147]. It provides data based on a variety of physical characteristics, such as size, morphology, surface texture, and roughness, in both qualitative and quantitative forms. It is also possible to determine statistical data such as size, surface area, and volume distributions. From 1 nm to 8 microns, a wide variety of particle sizes may be presented in the single scan. High resolution and visualization of 3D structures can be provided by AFM from the high-precision movement of the tip. Resolution in the vertical direction is constrained by the instrument’s vibration environment, but resolution in the horizontal direction is constrained by the diameter of the scanning tip. AFM instruments typically have X-Y resolutions of about 1 nm and vertical resolutions of less than 0.1 nm [148]. A cantilever with a sharp tip with a diameter of 10–20 nm is used to perform AFM. Si or Si_3_N_4_ is used in the microfabrication of AFM tips and cantilevers. When a laser beam is focused on the tip using a photodiode, the movement of the tip as a result of tip-surface interactions may be seen. The two primary modes of operation for AFM are contact and tapping modes [149]. In the tapping mode, the AFM cantilever is vibrated above the sample surface, causing the tip to come into touch with the surface only occasionally. In the contact mode, the AFM tip is continually in contact with the surface. Imaging in the tapping mode lessens shear pressures brought on by tip movement. Rao et al. used AFM in the dynamic mode to characterize the titanium oxide, zirconium oxide, and alumina nanoparticles and/or agglomerates on various surfaces [150]. The size, form, and distribution of TiO_2_, ZrO_2_ and alumina could be obtained. 

### 4.3. Other Important Characterizations

Auger electron spectroscopy (AES) is a surface-sensitive quantitative elemental analysis technique [151]. This method, which is similar to XPS, may be used to quantitatively identify all elements, with the exception of hydrogen and helium, within a depth of 2 nm, as well as provide some insight into the chemical state. The basic principle of AES is the Auger effect, in which a series of radiation-free transitions take place with an atom’s inner level being ionized. An Auger electron is ejected at the end of this process. L-level (auger electrons) electrons are expelled from the material by the irradiation of an electron beam following a sequence of electron transitions. AES benefits from having better spatial resolution (compared to the XPS). Researchers show evidence of using AES for nanomaterial characterization. For instance, Xu et al. used AES in determining the thickness of graphene films [115]. They reported that with more layers of graphene, the AES spectroscopy exhibits different spectral shape, intensity, and energetic features. This technique enables the precise and high-throughput measurement of up to six layer thicknesses of graphene as well as the detection of dopant and defect in graphene films on virtually any substrate. The availability of this trustworthy technique will enable direct examination of the mechanisms governing graphene formation as well as the investigation of unique features of graphene having different thicknesses on various substrates.

The Fourier transform infrared spectroscopy (FTIR) technique is a regularly used tool to identify surface functional groups and optical properties of samples in infrared spectral region. The FTIR spectra are usually recorded between 4000 cm^−1^ and 400 cm^−1^ [152]. FTIR is a technology that offers a lot of flexibility for the surface characterization of nanoparticles. Under certain conditions, it is feasible to determine the chemical composition of the surfaces of the NPs in addition to identifying the reactive surface sites that induce the surface reactivity. Using FTIR spectroscopy, various functional groups from the spectral bands are found to ascertain the conjugation between the nanomaterial and the adsorbed biomolecules [153]. The atoms in the nanoparticle vibrate in their bonds at frequencies that correspond to the absorption peaks in the FTIR spectrum. An FTIR spectrum is an essential information for qualitative research since the peak intensity gives a clear indication of the nature of the components present. Devaraj et al. used FTIR to identify the structure, associated bands, and bond stretching of the produced silver nanoparticles (AgNPs) from cannonball leaves [154]. 

**Figure 11:**
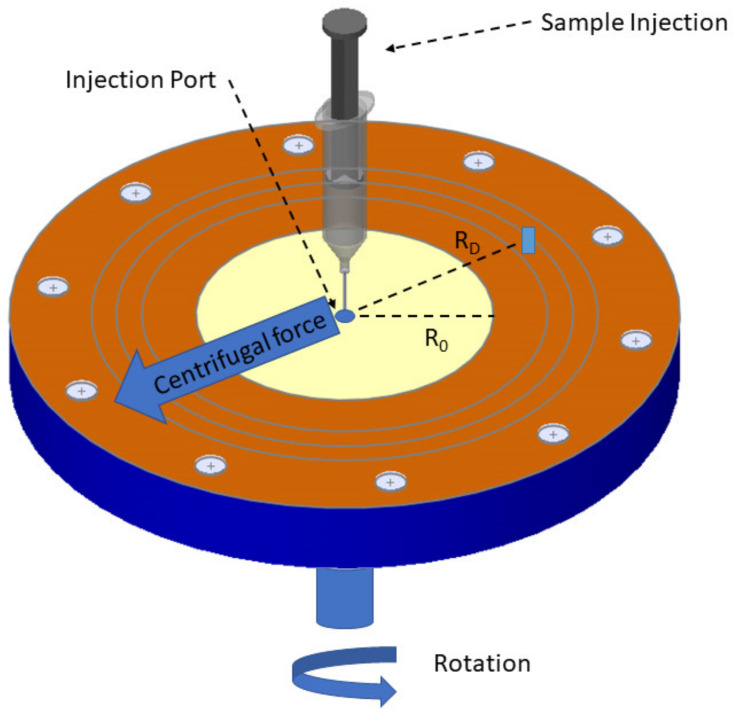
Schematic diagram of differential centrifugal sedimentation.

The benchtop method known as centrifugal photo-sedimentation (or differential centrifugal sedimentation, or DCS) is frequently employed to assess the high-resolution size distributions of NPs [106]. When subjected to a centrifugal force, DCS assesses how long it takes for nanoparticles (NPs) to sediment through a fluid, as shown in Figure 11. A prevalent issue in many naturally occurring and artificially created systems is agglomeration. For the grouping and read-across of nanoforms as well as for hazard and risk assessment of nanomaterials, reliable data on the agglomeration state are also essential. Clusters of nanoparticles with multimodal distributions in size, density, and shape are produced as a result of agglomeration. Although the technique is better recognized for particle size, these crucial variables have an impact on the sedimentation coefficient, which is the real physical quantity measured in DCS [155]. However, the assumption of spherical forms provides the foundation for the conversion into a particle size distribution. The latter ignores how the form itself affects the rate of sedimentation. Supra colloidal assemblies, often referred to as “colloidal molecules”, are used to illustrate how sedimentation coefficient distributions may be determined via DCS. 

Inductively coupled plasma mass spectrometry (ICP-MS), an atomic spectrometry method, has very low detection limits and can offer information for the majority of elements in the periodic table (noble gases H, N, O, F, and C are excluded). ICP-MS is thus one of the preferred methods for analyzing inorganic designed nanomaterials [156]. Increasingly, metallic nanoparticles (NP) at extremely low concentrations may be quantified and characterized using single particle inductively coupled plasma mass spectrometry (SP-ICP-MS). Viens et al. demonstrated that the quantification of ZnO nanoparticles is possible using ICP-MS [157]. The sensitive and selective technique known as single particle detection using inductively coupled plasma mass spectrometry allows for the direct analysis of individual nanoparticles in suspension and simultaneously provides data on size, size distribution, particle concentration, aggregation state, and ionic content [158]. 

The Brunauer–Emmett–Teller (BET) technique deals with the physio-sorption of gas molecules on a solid surface to quantify surface area, pore size, and pore volume of nanomaterial. Nitrogen gas is adopted for adsorption and desorption isotherms distribution determination. BET deviates from ideal analysis, as it considers multiple adsorption layers taking place in a realistic situation; therefore, this technique is also regarded as an extension of Langmuir adsorption, which only considers monolayer adsorption. However, the following assumptions are considered while working with the BET, as follows: (a) gas molecules tend to adsorbed physically on a solid surface in infinite layers, (b) gas molecules only interact with the adjacent layer, (c) Langmuir theory remains applicable to each layer, (d) adsorption enthalpy of the first layer remains constant, followed by enthalpy enhancement in subsequent layers, and (e) adsorption enthalpy of the second and subsequent layer is the same as liquefaction enthalpy. This process requires sample degassing through maintenance of particular temperatures, time, and vacuum conditions specific to the material under investigation. As an example, carbon quantum dots, CQDs, have enhanced surface area, which is beneficial for their performance in adsorption [159] and catalysis [160]. From BET analysis (surface area value > 1690 m^2^/g), it is evident that CQDs have a suitable position in adsorption applications owing to high surface area and polarity [159]. The synthesized composite photocatalyst TiO_2_/CQDs present a large surface area, which is verified by BET analysis, and is shown to augment pollutant concentration on nanocomposite surface, leading to the best photoactivity in the study [160]. In the case of carbon nanotubes (CNTs), an MWCNT-LVX antibacterial nano-conjugate was prepared via a facile synthesis route which showed minimized toxicity, high drug loading capacity, low effective dosage, and potent activity against the wound. The BET analysis confirms that the high surface area of CNTs allowed high drug loading efficiency by various interactions of LVX on the CNTs surface. The limitation of BET analysis lies in sole feasibility using dry powders, other than the enormous time required for characterization. For the graphene and its derived forms, Mohan et al. [161] observed that extent of BET surface area in different graphene derivatives varies; the highest SSA was observed for GNP with high structure uniformity and low defect density; lowest SSA was observed for dextrose reduced GO with poor structure orientation. 

Thermogravimetric analysis (TGA) is an analytical technique for quantitative and qualitative monitoring of mass loss or gains when the sample is heated, cooled, and held at a constant temperature atmosphere, either in stable or changing gas flow condition [162]. During analysis, the temperature is maintained, usually with following heating, cooling, isothermal maintenance, or their combination [163]. The TGA analyzer encloses the furnace and precise microbalance, which records sample weights in the closed furnace. It is usually used for assessing composition, thermal stability, or decomposition nature, the stoichiometry of chemical reactions, the kinetics of chemical reactions, adsorption or desorption processes, evaporation behavior, the influence of reactive gases, and evolved gas analysis. TGA technique has three categories, based on by-products generated or removed and changes in weight: dynamic TGA, static TGA, and quasi static TGA. It is employed by various industries ranging from pharmaceuticals to automotive industries. The TGA method is inexpensive, fast, and more straightforward for CQDs composition determination. In the synthesis of carbon dots-TiO_2_ nanosheets (CQDs -TNs), Li et al. [164] used TGA to determine the CQDs amount loaded on TNs. They observed that weight below 200 °C is due to adsorbed water evaporation, and weight loss between 200 °C and 700 °C is due to surface attached water molecule decomposition and combustion of CQDs. In the case of CNTs in bulk quantity [165], TGA can be used to assess population homogeneity, with essential parameters under consideration: initiation temperature, oxidation temperature, and residual mass. For graphene materials, Farivar et al. [166] established that TGA is a low-cost method for the quality control and characterization of manufactured graphene materials at an industrial scale. TGA is usually carried out with differential scanning calorimetry (DSC), as both techniques complement each other for successful material characterization. The non-equivalence of volatile mass loss and degradant formation is the main limitation of this method. 

UV-visible spectroscopy is an optical spectroscopic technique for the quantitative measurement of the light absorption property of a chemical substance present in a solution. A change in light intensity is determined by comparing light passing through a sample and another reference. It can make use of the Beer–Lambert law for concentration determination, providing information, such as sample composition and setup. This technique has many advantages, including non-destructive nature, direct, and easy operation, minimal pre-processing, and low cost. Yet, it could be affected by stray light, light scattering, and interferences from multiple light-absorbing species. For instance, CQDs usually possess excellent optical absorption in the UV region (260 to 320 nm), with the tail extending to the visible region. CQD absorption peaks come from π-π* aromatic sp^2^ domains transition and n-π* surface functional moieties (carbonyl, hydroxyl, ester, and carboxyl groups) transition, which further have a dependency on synthetic method and surface group nature [167]. As most CNTs are usually present in bundled form, they remain inactive in the UV-vis spectral region due to metallic and semiconducting CNTs tunnel coupling. In contrast, single CNTs have a detectable absorption peak in the UV-vis region [168,169]. CNT-water nanofluid stability was measured in terms of CNT concentrations as a function of time using UV-vis spectroscopy [170]. For graphene and its derivatives, spectra are observed in the 200–1000 nm range [171]. Graphene absorption features are attributed to surface plasmon resonance of free electrons and π electrons of carbonaceous material [171]. In the case of graphene oxide, the maximum absorption band is observed around 230 nm and up to 300 nm, which corresponds to π-π* transitions of C–C aromatic bonds. A shoulder at ~ 300 nm corresponds to n-π* transitions of C=O bonds [172,173,174]. Moreover, this technique can be employed to check graphene’s existence, functionality, and dispersed state in various solvents, such as 1-propanol, EtOH, ethylene glycol, tetrahydrofuran, and so on [175,176]. The only disadvantage of this method is the time consumption for sample analysis. 

Dynamic light scattering (DLS) is a non-invasive technique used for hydrodynamic particle size determination based on the measurement of the Brownian motion of particles in suspension, where smaller particles diffuse rapidly and larger particles diffuse slowly. The DLS instrument employs a laser to illuminate the suspended particle, followed by the use of the Stokes–Einstein equation for particle size, cluster size, and their distribution determination in a colloidal suspension. The particle size measurement depends on ionic strength, surface structure, and shape. In DLS measurement, the target solution placed in a cuvette is illuminated with laser, whose scattered photon is then counted and correlated for the final result. Dager et al. [177] used this method to study pristine CQD produced from Fennel seeds and found the average size to be approximately 6.1 nm. For CNTs, DLS can be used for size measurement with the Stokes–Einstein equation [178], while the hydrodynamic particle diameter and length of CNTs agglomerates in liquid solution can be determined [179,180,181]. For SiO_2_ nanoparticles dispersed in water, Prashant et al. used this method to understand particle size distribution and found that most particles were centered around 100 nm [182]. In the case of graphene nanosheet size determination, Amaro-Gahete et al. [183] investigated nanosheet size with the conjugation of laser doppler velocimetry method, and nanosheet size around 450 nm was found. Though DLS is a popular light scattering method for particle size determination, it suffers from a few limitations, such as dust particles interfering in photon correlation [184] and not being suitable for non-spherical particles [185]. 

Atomic absorption spectroscopy (AAS) is an analytical technique for measuring the metallic elements concentration of a sample by using electromagnetic wavelengths emanating from a light source. This technique relies on light absorption by free metallic ions to detect individual metallic particles. It also relies on the Beer–Lambert law. The quantification of metallic impurities of the sample becomes feasible through this analysis. AAS’s main components are an atomizer, radiation source, and spectrometer. It is capable of determination of over 70 different elements present in a sample. During MWCNT preparation, the used catalysts particles are removed by acidic treatment. Edwards et al. [186] demonstrated that usage of high-concentration acid leads to enhanced metallic impurities removal from purified CNTs. Pourjavid et al. [187] applied AAS with solid phase extraction to determine Mn(II), Fe(III), and Cu(II) ions in graphene oxide. Despite several advantages, AAS’s drawbacks are limited sensitivity, metallic-only samples, single-element detection capacity at a time, and limited linearity. 

Raman spectroscopy is a non-destructive, qualitative, and quantitative technique, which examines chemical structure, phase, polymorphy, crystallinity, and molecular interactions through the measurement of vibrational modes. In this technique, incidental light from the laser source falls on sample molecules, and then light interacts with chemical bonds and inelastic scattering, revealing its identity. In a Raman spectrum, each peak corresponds to a characteristic vibration mode of bonds, such as C–C, C–H, C=C, N–O, etc., and groups of bonds, such as benzene ring, polymer chain vibrations, etc. John et al. [167] used Raman spectroscopy to determine the crystallinity, optical, and electronic properties of CQDs. For CQDs, two characteristic peaks labeled D and G bands are observed around 135 cm^−1^ and 1585 cm^−1^ for sp^2^ hybridized graphitic bonds and sp^2^ bonded carbon atoms in E_2g_ mode of graphite hexagonal lattice, respectively [188]. For the structural integrity assessment of CNTs after acidic treatment, Raman spectroscopy can be used as it is susceptible to graphitic material structural disintegration [186,189]. In the case of graphene, Wang et al. [173] showed that nanosheets of graphene exhibited a strong D line at 1350 cm^−1^, corresponding to the breathing mode or Κ-point photons of A_1g_ symmetry, and a weak G line at 1580 cm^−1^, corresponding to the bond-stretching motion of in-plane pairs of C sp^2^ atoms. A huge merit of Raman spectroscopy is that it can be employed for all kinds of samples, such as solids, liquids, gels, slurries, and gases. However, owing to the insufficient strength of Raman scattering intensity, there exists a limit in terms of sensitivity and detection range for analyte concentration. The current limitation of basic Raman spectroscopy can be overcome by improved Raman techniques, such as resonance Raman spectroscopy and the use of an SERS platform.

## 5. Some Special Nanostructures

It is well-known that the shape and the morphology of the material can play a very crucial and significant role when it comes to the application. For example, a catalyst having dimensions in the nano/micro scale will possess a much greater surface area when compared to that of its bulk form. Therefore, the catalyst having dimensions in nano/micro scale will provide a greater number of active sites for the reaction to occur. In addition, if the surface area of contact is greater. Thus, it will provide more adhesion. To date, we have come across various techniques that can be used for the fabrication of nano/micro structures/arrays. This section will discuss some unique structures and their potential applications. 

### 5.1. Uniformly Spaced Mushroom Structures

Uniformly spaced mushroom structures find their applications mainly in the field of adhesion. The research area of strong adhesion is derived from multiple biological species found in nature. The science behind the adhesion is that most of the adhesive materials present in nature contain some elastic energy which easily follows the roughness that is present on the surface, which in turns increases the intimate contact between the adhesive agents and surface, resulting in the adhesion of two sides. However, it was also observed that the gecko feet (array of mushroom shaped) are on the contrary stiff and contain hairs which help the species to achieve adhesion [190]. 

Another similar example in nature are mushroom shaped micro structured attachment pads of beetles [191] (specially Chrysomelidae family) which provides adhesion to the species. Many researchers took inspiration from these natural species and tried to mimic the results in order to achieve strong adhesions. For example, in one of the research projects by Gorb et al., they bio-mimic the mushroom shaped fibrillar structures using polyvinyl siloxane and the template lithography process in order to achieve an adhesive structure [192]. Later, Carbone et al. explained that the high adhesive performance of a mushroom shaped micro/nano arrays over cylindrical arrays is due to the presence of a very thin annular plate attached to the top of the central pillar [193]. They also concluded that the presence of the non-uniformity of arrays over the surface can highly reduce the performance of the adhesive forces that occur between the microstructures and the surface. 

### 5.2. Semi-Circular Bumps

Transparent micro scale semi-circular bumps on a curved surface can find their application in the field of lens-on-lens type systems which can be used for optical imaging as such transparent microstructures provide a wide field of view and possess high light collecting capabilities. Inspiration of such systems arises from the structure of the insect’s eye. This type of microsystems can typically be formed by attaching a multiscale hierarchical structure superimposed over the primary structure. Obviously, fabricating such multistage regular primary and secondary uniform arrays over the substrate is a very tedious task.

Shao et al. proposed a fabrication method for such a semi-circular structure in order to mimic the morphology of insect eyes [194]. They proposed that if one intends to fabricate such a structure, one can use the nanoimprint lithography technique and laser beam swelling process for the secondary layer. They also suggested that the polymeric material should be used for the purpose of imprint lithography, and it must possess a desired amount of bulk flexibility after the curing process is over, so as to allow the underlying swelling polymeric material to expand in volume when the laser light is irradiated over the surface. 

### 5.3. Uniform Silver Coating

Development coating is one of the very interesting applications of nano/micro technology/fabrication. Coating on a surface not only prevents the surface from the attacks of foreign contaminants, but also enhances the functional features of the surface. Many works confirm that the material functionality of the surfaces totally changes only by changing the additive in the coating. A typical coating consists of a coating powder, binder polymeric matrix, volatile solvent, dispersant, and additives [195].

Silver being part of the noble metal family possesses excellent anti-microbial/bacterial properties. Hence, using silver as an additive can impart surface antibacterial and antimicrobial properties. For instance, Khalilabad et al. took multifunctional properties of silver to impart surface with antibacterial properties [196]. High density silver nanoparticles were fabricated/attached on the surface using the in-situ reduction of silver nitrate. The uniform silver nanoparticles were coated on fabric, and it was then reacted with hexadecyltrimethoxysilane (HDTMS) which surrounds the surface with an even coating of a low surface energy. Their results show that the prepared fabric shows antibacterial activities towards Gram-positive *Staphylococcus aureus* and Gram-negative *Escherichia coli*. 

### 5.4. Hierarchical Structures over Silicon Wafers

Nanopatterning on a silicon substrate has gained attention during the past decade. Silicon is one of the most abundant materials in the earth’s crust, so it is always readily available. The nanopatterning on the silicon surface can provide the surface with some of the high demanding properties, such as super hydrophobicity and high anti-reflection properties, which make the silicon surface suitable for its use in the fields of solar cell, photoluminescence, contamination prevention etc. 

Hierarchical structure fabrication over silicon wafer can be conducted using a few techniques. Bindra et al. used an extremely fast, electroless method to fabricate the hierarchical grooved cup shaped microstructures over the surface of silicon wafer. Later, silver pyramidal shape nanostructures were fabricated over these groves [197]. The final fabricated surface possesses antireflective properties which make the substrate useful in the field of optical devices. Cho et al. prepared hierarchical structures on a silicon wafer substrate using sacrificial layer methods [198]. It is noted that the nanostructures obtained using this sacrificial layer etching methods are denser, which results in the very low reflectance of the modified silicon surface. This sacrificial layer etching method is a one-step method as this layer can act as both micro- and nano-mask for the substrate by just varying the etching time. 

## 6. Applications

Advancements in the fields of nanotechnology have opened a limitless possibility in diverse fields of applications. This section will discuss some of the important applications for which nanomaterials can be used. 

### 6.1. Bio-Sensing and Bio-Imaging

The surface plasmon resonance is the exhibition of a resonance effect due to the interaction of free electrons of metal nanoparticles with incident photons. The inclusion of this property can find its applications for studying living body tissues where the specific molecule can be traced based on their interaction with metal nanoparticles inside a living body tissue [199]. For instance, researchers are using this approach to differentiate between a cancer and a healthy cell by coating the surface of the cancer cell. This goal is achieved by utilizing gold nanoparticles (AuNPs) which are coated on the antibodies with affinity towards cancer cells. Nonetheless the coupling between AuNPs and antibodies is evenly distributed among the healthy cells. The bio-imaging of these AuNPs coated antibodies with different morphologies are done at several wavelengths [200]. In another method, these AuNPs can be joined with proteins and other functional molecules instead of antibodies which later can be used for bio-imaging of cells. In a similar direction, cyclodextrins, porphyrins, supramolecules, polymers, and biomolecules, as well as other molecules that have antibodies, nucleic acids, and proteins, can also be used to identify and/or imitate the structure and function of natural occurring enzymes molecules. 

Research on nanozymes can be used in the areas of biosensors, immunoassays, stem cells growth, and environmental healing via pollution reduction/removal [201]. As discussed above, antibodies, proteins, and capsids can be used as a media for the identification of cancer-causing cells when self-assembling metallic NPs are coated over their surfaces. Along with that, alteration in naturally occurring proteins cells and antibodies with NPs [202,203] as well as individual synthetic proteins/antibodies [204,205] are attracting huge interest for their applications in the field of biomedicine and bio sensing. Moreover, hollow 3D bimolecular nanomaterials are highly beneficial for the efficient supply of targeted biomolecular drug deliveries, therapies, and diagnosis of complex diseases and genetic disorders [206]. Two-dimensional layered nanomaterials are another promising group for bio-applications. Transition-metal dichalcogenides and layered transition metal oxides attracted a lot of attention for biosensing and phototherapy [207,208]. For instance, 2D molybdenum trioxide (MoO_3_) nanosheets were employed to develop facile determination of some key analytes, such as glucose and hydrogen peroxide [209,210]. It was shown that the change in oxidation state of oxide nanosheets leads to a rapid bare eye detection probe for biological fluids [211]. 

### 6.2. Photonics and Optoelectronics

Modern optoelectronics and photonics industry contributes manifold essential products including optocouplers, solar harvesting cells, light-emitting diode (LED), laser diodes, photodetectors, Bragg reflectors, etc. [212]. Wide-band gap semiconductors, GaN and ZnO, are most important optoelectronic semiconductors for solid state lighting [213,214]. They have suitable direct energy band structure, proper band offset, efficient carrier transport, and stable chemical property for use in optoelectronic and electronic devices [215,216,217]. The commercial production of vital blue LEDs relies on metal-organic vapor phase epitaxy, in which epitaxial GaN thin film are grown on sapphire substrates. Due to the large mismatch in thermal expansion coefficient and lattice constants, dislocation occurs easily in this growth mode [218]. As bringing down dislocation density is important for the device performance, many strategies were proposed, such as the epitaxial lateral overgrowth (ELO) process and adoption of alternative substrates [219,220,221]. This kind of obstacle can be overcome by the use of one-dimensional semiconductor nanorods or nanorod-films [222]. It was found that ZnO nanorods prepared by the low-cost wet chemical approach have good luminescent property [223]. Besides, various *pn* junctions can be formed easily for LED or photodetector applications [224,225]. Due to the large exciton binding energy at room temperature (~60 meV) and versatile synthesis methods, ZnO nanorods are well-suited for optoelectronic devices [226,227]. 

For nanoscale semiconductors, the shape and size of material play an important role in electron–hole interactions within the core-shell type semiconductors. The past literature reported that a smaller diameter of holes is preferred instead of larger ones because it can significantly improve the performance of II-VI type semiconductor nanocrystals in case of optoelectronic applications [228]. In nanophotonics, nanoparticles can be used to convert near-infrared, visible, or ultraviolet photons into two, three, or four infrared photons with multi-photon quantum cutter [229]. In addition, monolayer transition-metal dichalcogenides (TMDs) coupled with conventional photonic crystals can provide material with new or enhanced optical functionalities that can be used to synthesize ultracompact photonic and optoelectronic devices. Ye et al. reported that a larger quantum yield of active material is crucial to achieve higher optical gain [230]. In their work, they experimentally measure the photoluminescence of MoS_2_, WS_2_, and WSe_2_ monolayers in order to optimize the thickness of TMD material for higher quantum yields. They reported that at cryogenic temperatures, monolayer WS_2_ provides a higher quantum yield which is in the order of five times higher than that of WSe_2_ and MoS_2_. 

### 6.3. Sensors

The interaction of living systems with surroundings is achieved via smell, taste, vision, hearing, and touch [231]. The demand for portable, economical, efficient, and sensitive chemical sensors for precision analysis has seen a rise in the past few decades. Major feature of sensors is that it has the potential to interconnect the physical, chemical, and biological worlds together [232]. In the case of sensors, the presence of a high area of interface between material and analyte plays a crucial role in defining the sensitivity, stability, and biocompatibility of the sensors [233]. Material–analyte interaction should be optimized in the case of sensors. It can be achieved by having a large, exposed surface area, a huge number of active sites that can effectively and selectively bind analytes, and the ability to convert binding events into a detectable signal. In addition, the material should have good structural and processing properties.

Many examples show that nanomaterials are well-suited to the manufacturing of gas, chemical, bio-chemical sensors. For instance, SnO_2_ nanosheets have been widely used for gas sensing [234]. In another study, Jiahua et al. successively utilized CNTs doped with SnO_2_ nanoparticles for the sensing of formaldehyde gas [235]. Liu et al. studied the electronic interactions and transport properties of various gas molecules, including CO, NH_3_, CO_2_, NO_2_, and NO molecules, on the surface of 2D monolayer graphene sensors [236]. 

In addition, 2D TMD materials also show promising potential for sensitive chemical sensing or biosensing [237]. For instance, MoSe_2_ in 2D nanosheet or 0D quantum dot forms can play the role of artificial enzyme to realize the colorimetric detection of hydrogen peroxide [238]. Furthermore, a luminescence probe for glucose was achieved for WS_2_ quantum dots, in which the oxidative effect on the rich edge states results in the luminescence quenching [239]. 

### 6.4. Energy Conversion and Storage 

Numerous limitations and shortfalls of fossil fuel are attributed to their non-renewable nature [240] and their doubtful availability for future generations to use in coming years [241]. Hence, there arises a need for the researchers and scientists to seek more renewable resources and readily available sources of energy. 

In contrast to fossil fuels [242], green energy production routes, including water splitting [243], electrochemical CO_2_ reduction [244,245], and piezoelectricity-based nano-generators [246], are better options to produce electrical energy [247]. As size being reduced to nanoscale dimensions [248], nanomaterials can preserve energy in various forms which makes them suitable for energy storage applications [247,249,250]. 

During photocatalytic reactions, photo-induced electrons and holes can trigger the production of reactive oxygen species, which in turn leads to hydrogen evolution reaction and oxygen reduction reaction [251]. These reactive species are also useful to eliminate contamination in our environment, such as organic dye molecules and residual antibiotic [252,253,254]. With broad surface area for reaction, proper oxidative potential, and efficient carrier transport, semiconductor nanomaterials are suitable for the low-cost production and storage of energy [255]. For instance, nanostructured TiO_2_ composite photo-anode with metal nanoparticles can be used for the production of energy via electrochemical water splitting using photo-electrochemical (PEC) mechanisms [256]. 

As an alternative, smart nano-energy harvesters subjected to certain external stimulus (temperature, electrical field, mechanical force, concentration gradient, etc.) and then respond to it with changes in their functionality. This can be thought of as a way to manufacture self-reliant devices. For instance, piezoelectricity based smart energy harvesters (PEH) can utilize mechanical energy to provide desirable electrical output, which provides another way to harvest energy. 

### 6.5. Functional Coatings

Functional coatings are sometimes called stimuli responsive coatings or smart coatings. These coatings dynamically change their physical or chemical properties when exposed to certain specific external stimulus which further alters the surface properties of material. These smart materials respond to several external stimuli, e.g., pressure, magnetic field, electric field, moisture, temperature, pH, acoustics etc. Several functional smart coatings, e.g., superhydrophobic, self-cleaning, anti-corrosive, antimicrobial, and antiviral coatings, are discussed below [257].

#### 6.5.1. Superhydrophobic Coatings

Over the past few decades, researchers have been trying hard to mimic the nature by inducing water repellent properties in a variety of material surfaces by coating them with materials with low surface energy. In nature a lot of plants, such as salvinia molesta, reed and lotus leaf, are famous for their superhydrophobic properties. These anti-wetting surfaces exhibit a water contact angle of more than 150° and sliding angle of less than 10°. Superhydrophobic surfaces can be used for multifarious applications, e.g., self-cleaning surfaces, anti-icing, anti-fogging, oil-water separation, and anti-corrosion. These coatings are fabricated by making the surface rough and further combining the surface with low surface energy materials, such as silanes, polymers, and fatty acids. A variety of methods are used to create the required surface roughness, such as depositing nanoparticles on the substrate or using different techniques to functionalize the surface. Zou et al. developed a dip coated superhydrophobic cotton surface using perfluoro-sulfonated polymer and carbon nanotubes [258]. The synthesized surface exhibited a water contact angle of 154.6° and demonstrated EMI shielding properties. Aminayi et al. used nanoparticle vapor deposition to acquire the surface roughness, followed by coating with tridecafluoro-1,1,2,2-tetrahydrooctyltrichlorosilane [259]. Trimethylaluminum-water nanoparticles were deposited on the surface which showed a contact angle of more than 160°. 

#### 6.5.2. Self-Cleaning Surfaces

Self-cleaning technology developed in the 20th century has a plethora of applications ranging from window glasses to solar panels. Self-cleaning is an interesting phenomenon which shows the removal of debris or dirt from the coating surface by the application of water on it. The design of these surfaces is derived from nature or living organisms. This is referred to as biomimetics or biomimicry. Lotus leaf, also known for its sacred purity in Asian countries for more than 2000 years, is the most studied self-cleaning surface. Koch et al. studied the surface morphology of the lotus surface and reported a water contact angle of 164° and contact angle hysteresis of about 3° [260]. Numerous artificial self-cleaning surfaces have been designed using nanomaterials, e.g., nano-silica, carbon nanotubes, and graphene. Jung and Bhushan fabricated a superhydrophobic surface using spray coating technique. The coating was composed of carbon nanotubes and epoxy resin with extremely low contact angle hysteresis [261]. Bravo et al. created a superhydrophobic film using silica nanoparticles and poly (allylamine hydrochloride) by LBL method [262]. The designed surface demonstrated a contact angle of about 160° and contact angle hysteresis of less than 10°.

#### 6.5.3. Anti-corrosive Coatings

The use of coatings has long been a traditional measure to protect metals from getting corroded. In the past few years, a large number of smart anticorrosive coatings have been synthesized using graphene. These coatings provide an excellent barrier between the substrate and the corrosive species present in the environment, suppressing corrosion advancement. Inorganic nano-containers, e.g., halloysite nanotubes (HNT), titanium dioxide nanotubes (TNTs), and mesoporous silica, can be used not only as corrosion inhibitors, but also as self-healing agents to repair the coating. Ubaid et al. studied TNTs loaded with epoxy monomer and dodecylamine (DDA) which act as corrosion inhibitor and self-healing agent [263]. The results from SEM analysis showed that the healing agents were able to repair the defects when the coating was damaged. These coatings form a barrier film on the metal surface and block the reaction taking place between the anodic and cathodic sites.

#### 6.5.4. Antimicrobial Coating

The global market for antimicrobial coatings has been valued at about 8.34 billion USD in the year 2020 and is expected to rapidly grow in the upcoming years. These coatings are used in products, such as door handles, push bars, touch screens, facemask, supermarket trolley, etc. The interest to fabricate such coatings reached an all-time high with the outbreak of global COVID-19 pandemic which has claimed the lives of more than 5 million people [264]. Silver nanoparticles with a large surface area have gained a lot of popularity to prevent bacterial infection. The silver nanoparticles are used in a variety of products like paints, food and textile industry, medical implants [265]. Zhao et al. investigated the antibacterial properties of Ag-TiN coatings fabricated using ion beam deposition method with a modulation period of 4–12 nm [266]. Meija et al. also investigated the combined effect of Ag/Cu nanoparticles on the TiAlN coatings for synthesizing antimicrobial surfaces [267]. They showed that incorporating the nanoparticles further enhanced its bactericidal effect against *E. coli* and *Staphylococcus aureus*. Zinc oxide nanoparticles are also the proven potent nanoparticles for antimicrobial applications. Esteban-Tejada et al. demonstrated the antifungal and antibacterial activity of ZnO nanoparticles coated transparent glasses [268]. The glass surface exhibited a chemical stability in different media and proved to be a potential inhibitor of *E. coli*, *S. aureus,* and *Candida krusei*. Additionally, superhydrophobic surfaces in the past few decades have gained significant attention to control the spread of infectious bacteria by minimizing the contact area between the substrate and microbes. Hizal et al. designed a nano-pillared hydrophobic surface on the aluminum surface and further coated it with low surface energy Teflon [269]. The synthesized surface showed a significant reduction to the adhesion of microorganisms, e.g., *E. coli* and *S. aureus*. Bartlet et al. prepared a superhydrophobic titanium surface by creating titania nanotube arrays using a chemical etching and anodizing technique [270]. The developed surface resulted in reduced bacterial adhesion on the surface. 

### 6.6. Drug Delivery System

The advent of nanotechnology has led to the development of nanomedicines or nanoparticle-based drugs, offering a large variety of opportunities to diagnose, treat, and cure diseases such as cancers. Nanoparticles designed for such applications are prepared with various biological and chemical routes which are inexpensive and eco-friendly. The nanoscale size of these entities allows them to easily diffuse into the cell, further interacting with specific cellular components. The nanomedicines permit selective targeting, decrease the drug-related toxicity, and promote the patient’s compliance with less frequent dosing. These particles are designed in such a manner that they are compatible with the immune system (non-immunogenic). Several advantages of these nano drugs are listed below.

- Increased surface area for drug dose;
- Smaller dosage requirement;
- Increased dissolution rate.
- Promotes rapid therapeutic action;
- Increased solubility;

Different types of nanostructures have been used to increase the efficiency of drug delivery to specific targets. Metallic nanoparticles can incorporate a higher drug dose due to the higher surface area. The gadolinium nanoparticles modified with thiamine, folate, and polyethylene glycol have been studied for tumor targeted drug delivery. Liu et al. utilized the self-assembly of PBE-containing block copolymer poly (ethylene glycol)-block-poly (amino phenylboronic ester) (PEG-b-PAPBE), encapsulating GO_x_ in the micelles, for an insulin delivery system with dual responsiveness to glucose and H_2_O_2_ [271]. Polymeric nanoparticles, e.g., polycaprolactone, polyacrylate, alginate, collagen, and albumin, can deliver drugs with minimum level of toxicity and are characterized by more specific drug targeting and delivery. Abraxane, which is an albumin-based paclitaxel nanoparticle, is used in the treatment of breast cancer. Nanotechnology for medicine has proven to be very effective and has opened up new opportunities to provide customizable, safer, and more effective treatment. Rapid progress is predicted in the field of nanobiotechnology in upcoming years. 

## 7. Highlighted Examples

Even though there is a wide range of possible applications in which many nanomaterials are promising for use, some of them have received particular attention nowadays. In addition, some types of nanomaterials are also employed rather frequently, such as TiO_2_, ZnO, graphene, and MoS_2_. This section will provide a few examples, highlighting the interplay between these “hot” nanomaterials and several applications urgently in need for our society. 

### 7.1. Photocatalysis

**Figure 12:**
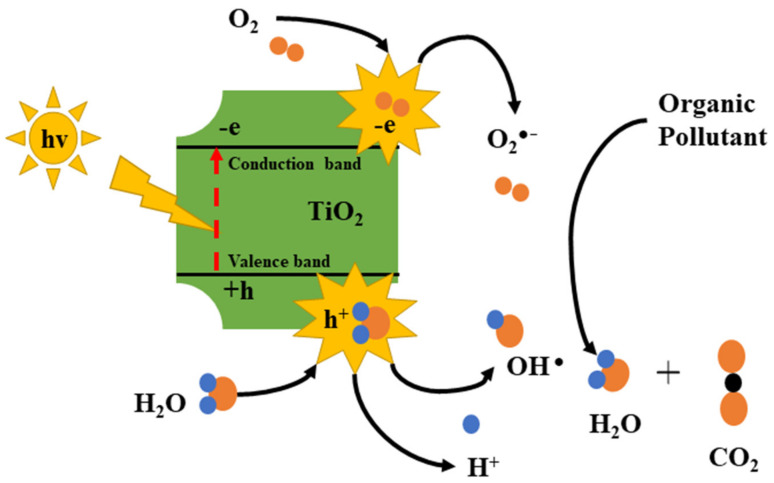
Illustration of basic photodegradation processes using TiO_2_ photocatalyst.

Photocatalytic H_2_ production from water splitting using solar-driven semiconductor is an attractive technology to convert solar energy into chemical energy. In the field of wastewater treatment, photocatalytic degradation of harmful organic molecules using semiconductor photocatalysts is also a promising approach to solve the problems of environmental pollution. TiO_2_ is known as a traditional semiconductor photocatalyst with many merits, including good photoresponse, excellent chemical stability, low price, non-toxicity, and environmentally friendly nature. It is commonly used to promote the decomposition of toxic and harmful organic pollutants to reduce environmental damage. In general, the basic photocatalytic degradation activity of TiO_2_ can be explained by the charge carrier generation and reactive species formation, as illustrated in Figure 12. Because TiO_2_ has a wide band gap (3.2 eV), it can only absorb 4% of the ultraviolet (UV) light in the entire solar spectrum. To make use of visible light, which accounts for 45% of the total energy in natural light, researchers developed a lot of means to overcome the limitation of the wide band gap, such as doping and co-catalyst loading. Zhou et al. adopted MoS_2_ as a cocatalyst to load on TiO_2_ nanobelt for photocatalytic hydrogen production [272]. They used the hydrothermal method to coat a few layers of MoS_2_ nanosheet on TiO_2_ nanobelt with rough surfaces, forming a 3D hierarchical core-shell structure. They show that the synthesized TiO_2_@MoS_2_ heterostructure is an excellent photocatalyst for highly efficient and stable hydrogen production without the Pt co-catalyst. In addition, the TiO_2_@MoS_2_ heterostructure also exhibited excellent performance in the adsorption and photocatalytic decomposition of organic dyes. Synergistic effects can be attributed to the matched energy band of TiO_2_/MoS_2_ heterointerface, which favors the charge transfer and suppresses the photoelectron-hole recombination between MoS_2_ and TiO_2_, leading to the enhanced photocatalytic activity.

**Figure 13:**
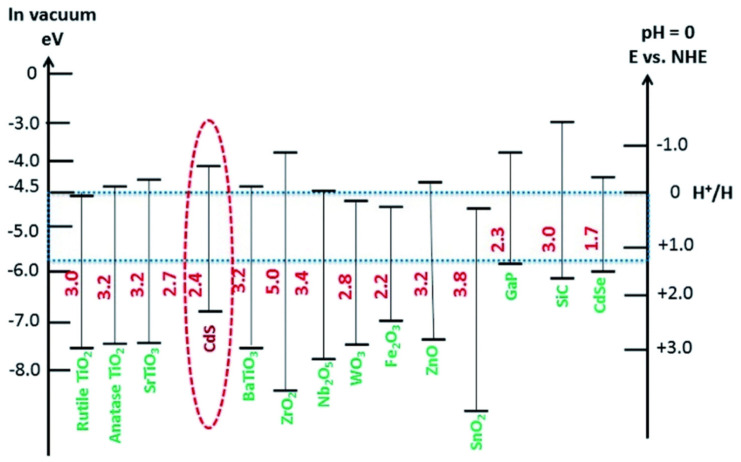
Schematic diagram of energy level positions for CdS and other semiconductors relative to the redox potential of water. Reproduced with permission from [273]. Royal Society of Chemistry, 2020.

In contrast to TiO_2_, cadmium sulfide (CdS) has a narrow band gap of 2.4 eV at room temperature, which allows for highly efficient absorption of visible light. As shown in Figure 13, CdS nanomaterials possess adequate energy levels for multiple photocatalytic reactions, including CO_2_ reduction, hydrogen production, and organic dye degradation [273]. However, there still exist a few drawbacks of pure CdS materials for use as semiconductor photocatalyst. For example, CdS materials suffer serious photo-corrosion during photocatalysis because the sulfide anions can be easily oxidized by photo-generated holes, making the CdS materials rather unstable. Besides, CdS nanomaterials generally exhibit lower adsorption capacity for reactants and easily undergo aggregation during reaction, which may reduce their surface areas and enhance the recombination rate of photo-generated carriers, suppressing the overall photocatalytic activity. To meet the requirements of practical applications, it is possible to mitigate the shortcomings by tuning the morphology of CdS nanomaterials or by the incorporation of a sacrificial reagent material. In this regard, MoS_2_ can also be used as a co-catalyst to strengthen the photocatalytic performance of CdS-based nanomaterials. Liu et al. reported a CdS/MoS_2_ nanocomposite synthesized by hydrothermal method, which was then characterized by SEM, TEM, XRD, and XPS [274]. The obtained nanocomposite shows a unique nanostructure with dendritic CdS branched nanowires as the scaffold and MoS_2_ coated on the surface of the dendritic structure. The formation of a heterojunction between the CdS branch nanowires and amorphous MoS_2_ layer facilitates the separation and transfer of photo-generated charge carriers. The photocatalytic oxidation–reduction reaction can be promoted by the matched energy levels of the coupled semiconductor materials. The MoS_2_ layer on the surface provides a large number of active sites on sulfur edges. In addition, it was found that the MoS_2_ layer can also act as a sacrificial agent to inhibit CdS corrosion. These advantageous factors eventually enhance the photocatalytic activity, stability, and reusability of the hybrid nanomaterial, compared to pure CdS nanomaterials.

As a semiconductor material with appropriate band gap for visible-light absorption (1.9 eV), MoS_2_ is also noted in the field of semiconductor photocatalyst. However, the problem of short lifetime of photo-induced carrier still needs to be overcome. Islam et al. devised a two-stage cost-efficient synthesis protocol of quasi-0D/2D ZnO/MoS_2_ nanocomposites [253]. It combines a “top-down” liquid exfoliation approach and a “bottom-up” wet chemical process to deposit quasi-0D ZnO nanoparticles on atomically thin molybdenum disulfide 2D nanosheets. The intimate contact between MoS_2_ nanosheets and ZnO NPs forms n-n type heterojunction, which facilitates interfacial carrier transfer and separation, leading to enhanced photocatalytic activity. They demonstrated that the obtained heterodimensional ZnO/MoS_2_ nanocomposites not only degrade typical organic dyes under visible-light irradiation, but also decompose a commonly used antibiotic, tetracycline, which shows great potential for environmental remediation. Besides, this low-cost strategy can be extended to hybridize other 2D TMD nanosheets with various semiconductor quantum dots to rationally design a composite semiconductor photocatalyst.

### 7.2. Batteries

Traditional energy from fossil fuel is no longer suitable for the sustainable economy. Concern for energy crisis is serious for our society. Energy storage technologies with high energy density and long cycle life are vital for renewable energy storage. In our daily life, rechargeable batteries are used in various way, such as a portable electronic device, a power tool, or an electric vehicle. The exploration of various electrochemical devices for metal-ion batteries has received a lot of attention [275]. Carbon nanomaterials with graphitic structure, such as few-layered graphene, remains a tempting choice for battery application. However, it is very difficult to have large quantities of high-quality graphene by popular preparation methods such as exfoliation and epitaxial approaches. While the former usually suffers from high density of structural defects and chemical residues, the latter is neither cost-efficient nor likely for large-scale industrial production. Therefore, the cost-effective and large-scale production of high-quality graphene remains a prime challenge for the success of graphene-based batteries. Recently, Zhang et al. reported a low-cost sulfur-assisted synthesis method, which converts benzene rings of tetraphenyltin into high purity crystalline graphene [276]. This method enables the large-scale synthesis of three-dimensional microspheres consisting of a few layers graphene. It shows excellent electron mobility, as expected. Moreover, the unique 3D morphology of graphene microstructure mitigated the high inter-sheet junction contact resistance, leading to the enhanced performance of graphene electrodes. It was shown that the graphene microsphere-based anodes in potassium-ion batteries exhibited a low discharge platform (average discharge platform about 0.1 V), a high initial capacity, a high rate performance, and excellent cycling stability. Similarly, the graphene microsphere-based electrodes also enhanced the performance of lithium-ion and aluminum batteries.

### 7.3. Electrocatalysis

**Figure 14:**
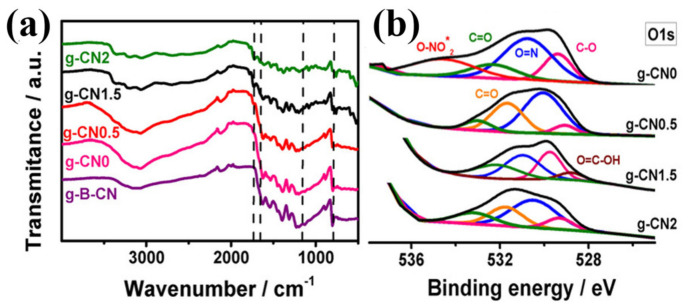
(**a**) The surface oxygen functional group of g-C_3_N_4_ nanorods can be evidenced by the appearance of the peak at 1780 cm^−1^ which is due to the stretching and bending vibrations of carbonyl and carboxyl groups. (**b**) The high-resolution XPS spectra of O-1s with its deconvolution clearly reveal the presence of C=O and C−O. Reproduced with permission from [277]. John Wiley and Sons, 2019.

Hydrogen is regarded as one of the most promising sources of clean energy. Nevertheless, the high energy demand associated with one of the half-cell reactions, namely oxygen evolution reaction (OER) at the anode remains a bottleneck for the efficiency of the overall process. Thus, a lot of effort has been made to search for a robust and proficient electrocatalyst for OER with low cost. Metal-free carbon-based materials with high conductivity appears to be nice candidates for OER catalysts. It was known that the incorporation of electronegative heteroatoms, such as N and B, can improve the catalytic activity of carbon-based materials. Graphitic carbon nitride (g-C_3_N_4_) could be regarded as a material derived from introducing high N content into carbon materials. Thus, it can be a promising candidate in this regard. Desalegn et al. reported the preparation of g-C_3_N_4_ nanorods by a facile two step synthesis route [277]. Urea was used as a structure directing agent to tailor the morphology. The OER catalytic activity of synthesized electrocatalysts with carbon fiber cloth substrate was investigated in 1 M KOH aqueous solution. They showed that the 1D g-C_3_N_4_ electrocatalysts present a low overpotential of 316 mV at 10 mA cm^−2^, which is among the lowest reported overpotential for carbon-based electrocatalysts for the OER. It also demonstrates a robust activity in an alkaline electrolyte for 24 h by retaining 100 % of its initial current density. The excellent electrocatalytic performance can be attributed to a few factors. The 1D nanorod structure is beneficial for charge transport and transfer to exposed active sites. Besides, the nanorod morphology is efficient for the desorption of evolved gas from the electrode surface such that the active sites are free for reactions. It was known that N doped carbon has a lot of C−N bonds on edge and defective sites, which can make nearby carbon atoms positively charged. These carbon atoms serve as adsorption sites for OH^−^ or H_2_O via electrostatic forces. In turn, electron transfer between catalyst surfaces and reaction intermediates, such as O_2_^2−^ and O^2−^, is enhanced, leading to an efficient OER process. Moreover, FTIR and XPS measurements show that g-C_3_N_4_ nanorods possess a number of oxygen containing functional groups, as shown by Figure 14 [277]. These functional groups can make adjacent carbon atoms positively charged because of the electron withdrawing oxygen atoms. They can be other active sites to expedite oxygen evolution, so a dual active site (C−N and C=O/C−O) mechanism for OER can be responsible for the lower overpotential. In short, the co-existence of nitrogen and oxygen in the catalyst is significant for the formation of dual active sites as an efficient electrocatalyst for the OER activity.

## 8. Conclusions and Future Outlook

In the past few decades, the quest for low-dimensional nanomaterials was usually followed by subsequent innovations in nanotechnology and nanoscience. Semiconductor nanostructures are endowed with an enriched surface area, a high surface-to-volume ratio, affluent surface/interface effects, and distinctive physical and chemical properties due to size quantization, resulting in new capabilities and favorable synergistic effects for their widespread applications. This review article begins with a brief history of nanomaterials and nanotechnology. Nanomaterials were categorized according to their degree of spatial confinement in the quantum limit. Nanofabrication paves the way for realization of desired nanostructures and functionalities. Essential nanofabrication techniques in both bottom-up and top-down approaches were presented expansively. Various characterization techniques for low-dimensional semiconductors were then introduced. The final section addresses significant applications of nanomaterials, including photonics, sensors, catalysis, energy storage, coatings, and various bio-applications. In the near future, significant breakthroughs in nanotechnology and nanoscience will continue to depend on the advancement of low-dimensional nanomaterials. For instance, challenges in the large-area CVD production of 2D TMD materials remains a bottleneck for next-generation electronics. Compatible processing techniques with current Si-based technology also require further investigations. In contrast, low-dimensional semiconductor nanocomposites would reach their commercial market much faster in many other fields, such as sensors, coatings, energy storage, and conversion.

## Figures and Tables

**Table 1:** Advantages of “Top-down” and “Bottom-up” techniques for nanofabrication.

Top-Down Approach	Bottom-Up Approach
Fabrication over a large-scale substrate is possible	Ultra-fine nanoparticles, nano shells, nanotubes, nano arrays can be prepared.
Process of chemical purification is not required	Deposition parameters can be easily controlled by varying reaction temperature, precursor concentration, etc.
	Comparatively cheaper technique.

**Table 2:** Disadvantages of “Top-down” and “Bottom-up” techniques for nanofabrication.

Top-Down Approach	Bottom-Up Approach
Size distribution is broad (10–100 nm).	Scale-up of the process is difficult.
Control over deposition parameters is tedious.	Chemical purification of final product is required.
Often expensive technique.	

**Table 3:** Summary and comparison of different characterization techniques.

S. No.	Characterization Technique	Principle	Entity Identified	Reference
1	X-ray Diffraction (XRD)	Constructive interference of monochromatic X-ray	Size (structural properties), elemental-chemical composition, crystal structure	[96]
2	X-ray Absorption Spectroscopy (XAS)	X-ray absorption and fluorescence, Multiple Scattering	Chemical state, oxidation state	[97]
3	Small Angle X-ray Scattering (SAXS)	Collisions between an incoming wave and a surface particle results in wave scattering in all direction	Size (structural properties), size distribution, growth kinetics	[98]
4	X-ray Photoelectron Spectroscopy (XPS)	Electrons within a sample absorb photons of a particular energy and then emerge from the solid	Ligand binding, surface composition, chemical state-oxidation state, Elemental-chemical composition	[99]
5	Fourier Transform Infrared Spectroscopy (FTIR)	Gases absorb IR radiation at species-specific frequencies	Ligand binding, composition, density, arrangement, mass, surface composition	[100]
6	Nuclear Magnetic Resonance (NMR) Spectroscopy	Transfer of energy is possible from base energy to higher energy levels when an external magnetic field is applied	Size (structural properties), elemental-chemical composition, crystal structure, growth kinetics, ligand binding, composition, density, arrangement, mass, surface composition, surface area, specific surface area	[101]
7	Brunauer-Emmett-Teller (BET)	Physical adsorption of gas molecules on a solid surface	Surface area, specific surface area	[102]
8	Thermogravimetric Analysis (TGA)	Dynamic relationship between temperature with a change in physical property like mass change or enthalpy change etc.	Ligand binding, composition, density, arrangement, mass, surface composition	[103]
9	UV-visible Spectroscopy	Absorption of ultraviolet light or visible light by materials	Optical properties, concentration, size (structural properties)	[104]
10	Dynamic Light Scattering (DLS)	Brownian motion of macromolecules in solution that arises due to bombardment from solvent molecules	Size (structural properties), size distribution, agglomeration state	[105]
11	Differential Centrifugal Sedimentation (DCS)	Time taken by nanoparticles to sediment through a fluid when exposed to centrifugal field	Size (structural properties), size distribution, agglomeration state, density	[106]
12	Inductively Coupled Plasma Mass Spectrometry (ICP-MS)	Ions of analyte atoms are sorted and quantified based on their mass-to-charge ratio	Size (structural properties), elemental-chemical composition, size distribution, concentration, single particle properties	[107]
13	Transmission Electron Spectroscopy (TEM)	Beam of electrons is transmitted through a specimen to form an image	Structural defects, size, shape, 3D visualization, single particle properties, agglomeration state	[108]
14	Scanning Electron Microscopy (SEM)	Beam of secondary electrons emitted from a specimen to form an image	Size (structural properties), size distribution, 3D visualization, NPs detection	[109]
15	Scanning Transmission Electron microscopy (STEM)	Properties of both SEM and TEM	Crystal structure, NPs detection, optical properties	[110]
16	Energy Dispersive X-Ray (EDX)	Generation of characteristic X-rays from a specimen through the electron beam	Elemental-chemical composition	[111]
17	Atomic Force Microscopy (AFM)	Surface interaction between sharp tip and surface atoms	Structural defects, size, shape, 3D visualization	[112]
18	Auger Electron Spectroscopy (AES)	Auger effects: a chain of radiation-less transitions in an atom in which one of its inner levels is ionized	Elemental-chemical composition, thickness	[113,114,115]
19	Atomic Absorption Spectroscopy (AAS)	Atoms (and ions) can absorb light at a specific, unique wavelength	NPs detection, elemental-chemical composition	[116,117]
20	Raman Spectroscopy	Inelastic scattering of laser light	Fingerprint of chemical species, bonding nature	[118]

## Data Availability

Not applicable.

## References

[1] Kolahalam L.A., Kasi Viswanath I.V., Diwakar B.S., Govindh B., Reddy V., Murthy Y.L.N. (2019). Review on Nanomaterials: Synthesis and Applications. Mater. Today Proc..

[2] Baig N., Kammakakam I., Falath W., Kammakakam I. (2021). Nanomaterials: A Review of Synthesis Methods, Properties, Recent Progress, and Challenges. Mater. Adv..

[3] Vollath D. (2013). Nanomaterials: An Introduction to Synthesis, Properties and Applications.

[4] Haghanifar S., Tomasovic L.M., Galante A.J., Pekker D., Leu P.W. (2019). Stain-Resistant, Superomniphobic Flexible Optical Plastics Based on Nano-Enoki Mushroom-like Structures. J. Mater. Chem. A.

[5] David N., Djilali N., Wild P. (2012). Fiber Bragg Grating Sensor for Two-Phase Flow in Microchannels. Microfluid. Nanofluidics.

[6] Qi D., Lu N., Xu H., Yang B., Huang C., Xu M., Gao L., Wang Z., Chi L. (2009). Simple Approach to Wafer-Scale Self-Cleaning Antireflective Silicon Surfaces. Langmuir.

[7] Gao Y., Peng Z., Shi T., Tan X., Zhang D., Huang Q., Zou C., Liao G. (2015). Bio-Inspired Fabrication of Complex Hierarchical Structure in Silicon. J. Nanosci. Nanotechnol..

[8] Dao T.C., Luong T.Q.N. (2020). Fabrication of Uniform Arrays of Silver Nanoparticles on Silicon by Electrodeposition in Ethanol Solution and Their Use in SERS Detection of Difenoconazole Pesticide. RSC Adv..

[9] Kang S.M., Kim H., Choi J.S., An J.H. (2021). Bioinspired Omniphobic Microchamber Structure. Adv. Mater. Interfaces.

[10] Tiwari J.N., Tiwari R.N., Kim K.S. (2012). Zero-Dimensional, One-Dimensional, Two-Dimensional and Three-Dimensional Nanostructured Materials for Advanced Electrochemical Energy Devices. Prog. Mater. Sci..

[11] Wang Z., Hu T., Liang R., Wei M. (2020). Application of Zero-Dimensional Nanomaterials in Biosensing. Front. Chem..

[12] Pandit S., Behera P., Sahoo J., De M. (2019). In Situ Synthesis of Amino Acid Functionalized Carbon Dots with Tunable Properties and Their Biological Applications. ACS Appl. Bio. Mater..

[13] Yan Y., Gong J., Chen J., Zeng Z., Huang W., Pu K., Liu J., Chen P. (2019). Recent Advances on Graphene Quantum Dots: From Chemistry and Physics to Applications. Adv. Mater..

[14] Zhang C., He J., Zhang Y., Chen J., Zhao Y., Niu Y., Yu C. (2018). Cerium Dioxide-Doped Carboxyl Fullerene as Novel Nanoprobe and Catalyst in Electrochemical Biosensor for Amperometric Detection of the CYP2C19*2 Allele in Human Serum. Biosens. Bioelectron..

[15] Robidillo C.J.T., Wandelt S., Dalangin R., Zhang L., Yu H., Meldrum A., Campbell R.E., Veinot J.G.C. (2019). Ratiometric Detection of Nerve Agents by Coupling Complementary Properties of Silicon-Based Quantum Dots and Green Fluorescent Protein. ACS Appl. Mater. Interfaces.

[16] Ou J., Tan H., Chen Z., Chen X. (2019). FRET-Based Semiconducting Polymer Dots for PH Sensing. Sensors.

[17] Bagheri N., Khataee A., Habibi B., Hassanzadeh J. (2018). Mimetic Ag Nanoparticle/Zn-Based MOF Nanocomposite (AgNPs@ZnMOF) Capped with Molecularly Imprinted Polymer for the Selective Detection of Patulin. Talanta.

[18] Sondhi P., Maruf M.H.U., Stine K.J. (2019). Nanomaterials for Biosensing Lipopolysaccharide. Biosensors.

[19] Shi X., Meng H., Sun Y., Qu L., Lin Y., Li Z., Du D. (2019). Far-Red to Near-Infrared Carbon Dots: Preparation and Applications in Biotechnology. Small.

[20] Panwar N., Soehartono A.M., Chan K.K., Zeng S., Xu G., Qu J., Coquet P., Yong K.-T., Chen X. (2019). Nanocarbons for Biology and Medicine: Sensing, Imaging, and Drug Delivery. Chem. Rev..

[21] Molaei M.J. (2019). Carbon Quantum Dots and Their Biomedical and Therapeutic Applications: A Review. RSC Adv..

[22] Su B., Wu Y., Jiang L. (2012). The Art of Aligning One-Dimensional (1D) Nanostructures. Chem. Soc. Rev..

[23] Gholami T., Pirsaheb M. (2021). Review on Effective Parameters in Electrochemical Hydrogen Storage. Int. J. Hydrog. Energy.

[24] Cardoso G.L., Piquini P.C., Khossossi N., Ahuja R. (2021). Lithium-Functionalized Boron Phosphide Nanotubes (BPNTs) as an Efficient Hydrogen Storage Carrier. Int. J. Hydrogen Energy.

[25] Sharma A. (2020). Investigation on Platinum Loaded Multi-Walled Carbon Nanotubes for Hydrogen Storage Applications. Int. J. Hydrog. Energy.

[26] Huang T., Huang X., Hu C., Wang J., Liu H., Ma Z., Zou J., Ding W. (2021). Enhancing Hydrogen Storage Properties of MgH_2_ through Addition of Ni/CoMoO_4_ Nanorods. Mater. Today Energy.

[27] Novoselov K.S., Geim A.K., Morozov S.V., Jiang D., Zhang Y., Dubonos S.V., Grigorieva I.V., Firsov A.A. (2004). Electric field effect in atomically thin carbon films. Science.

[28] Novoselov K.S., Geim A.K., Morozov S.V., Jiang D., Katsnelson M.I., Grigorieva I., Dubonos S.V., Firsov A. (2005). Two-dimensional gas of massless Dirac fermions in graphene. Nature.

[29] Singh V., Joung D., Zhai L., Das S., Khondaker S.I., Seal S. (2011). Graphene based materials: Past, present and future. Prog. Mater Sci..

[30] Fatima J., Shah A.N., Tahir M.B., Mehmood T., Shah A.A., Tanveer M., Nazir R., Jan B.L., Alansi S. (2022). Tunable 2D Nanomaterials; Their Key Roles and Mechanisms in Water Purification and Monitoring. Front. Environ. Sci..

[31] Chhowalla M., Jena D., Zhang H. (2016). Two-Dimensional Semiconductors for Transistors. Nat. Rev. Mater..

[32] Hu T., Mei X., Wang Y., Weng X., Liang R., Wei M. (2019). Two-Dimensional Nanomaterials: Fascinating Materials in Biomedical Field. Sci. Bull..

[33] Tan C., Cao X., Wu X.-J., He Q., Yang J., Zhang X., Chen J., Zhao W., Han S., Nam G.-H. (2017). Recent Advances in Ultrathin Two-Dimensional Nanomaterials. Chem. Rev..

[34] Khan A.H., Ghosh S., Pradhan B., Dalui A., Shrestha L.K., Acharya S., Ariga K. (2017). Two-Dimensional (2D) Nanomaterials towards Electrochemical Nanoarchitectonics in Energy-Related Applications. Bull. Chem. Soc. Jpn..

[35] Fang Z., Xing Q., Fernandez D., Zhang X., Yu G. (2020). A Mini Review on Two-Dimensional Nanomaterial Assembly. Nano Res..

[36] Hang D.R., Huang C.F., Cheng K.A. (2009). Probing semiclassical magneto-oscillations in the low-field quantum Hall effect. Phys. Rev. B.

[37] Paramasivam G., Palem V.V., Sundaram T., Sundaram V., Kishore S.C., Bellucci S. (2021). Nanomaterials: Synthesis and Applications in Theranostics. Nanomaterials.

[38] Wang K., Ma Q., Qu C.-X., Zhou H.-T., Cao M., Wang S.-D. (2022). Review on 3D Fabrication at Nanoscale. Autex Res. J..

[39] Iqbal P., Preece J.A., Mendes P.M. (2012). Nanotechnology: The “Top-Down” and “Bottom-Up” Approaches. Supramol. Chem..

[40] Teo B.K., Sun X.H. (2006). From Top-down to Bottom-up to Hybrid Nanotechnologies: Road to Nanodevices. J. Clust. Sci..

[41] Ochekpe N.A., Olorunfemi P.O., Ngwuluka N.C. (2009). Nanotechnology and Drug Delivery. Part 1: Background and Applications. Trop. J. Pharm. Res..

[42] Thirumalai J., Thirumalai J. (2018). Introductory Chapter: The Eminence of Lithography—New Horizons of Next-Generation Lithography. Micro/Nanolithography: A Heuristic Aspect on the Enduring Technology.

[43] Wu W., Dey D., Memis O.G., Katsnelson A., Mohseni H. (2008). Fabrication of Large Area Periodic Nanostructures Using Nanosphere Photolithography. Nanoscale Res. Lett..

[44] Francioso L., Siciliano P. (2006). Top-down Contact Lithography Fabrication of a TiO_2_ Nanowire Array over a SiO_2_ Mesa. Nanotechnology.

[45] Kim K., Park K., Nam H., Kim G.H., Hong S.K., Kim S., Woo H., Yoon S., Kim J.H., Lim G. (2021). Fabrication of Oblique Submicron-Scale Structures Using Synchrotron Hard X-ray Lithography. Polymers.

[46] Menumerov E., Golze S.D., Hughes R.A., Neretina S. (2018). Arrays of Highly Complex Noble Metal Nanostructures Using Nanoimprint Lithography in Combination with Liquid-Phase Epitaxy. Nanoscale.

[47] Jang H.I., Yoon H.S., Lee T.I., Lee S., Kim T.S., Shim J., Park J.H. (2020). Creation of Curved Nanostructures Using Soft-Materials-Derived Lithography. Nanomaterials.

[48] Howell S.T., Grushina A., Holzner F., Brugger J. (2020). Thermal Scanning Probe Lithography—A Review. Microsyst. Nanoeng..

[49] Córdoba R., Orús P., Strohauer S., Torres T.E., De Teresa J.M. (2019). Ultra-Fast Direct Growth of Metallic Micro- and Nano-Structures by Focused Ion Beam Irradiation. Sci. Rep..

[50] Yoon G., Kim I., So S., Mun J., Kim M., Rho J. (2017). Fabrication of Three-Dimensional Suspended, Interlayered and Hierarchical Nanostructures by Accuracy-Improved Electron Beam Lithography Overlay. Sci. Rep..

[51] Ishihara T., Luo X., Kawata S., Masuhara H. (2006). Chapter 17 Nanophotolithography Based on Surface Plasmon Interference: Handai NanoPhotonics.

[52] Tanaka H., Thomas F.K. (2015). Epitaxial Growth of Oxide Films and Nanostructures. Handbook of Crystal Growth.

[53] Toropov N., Vartanyan T. (2019). Noble Metal Nanoparticles: Synthesis and Optical Properties.

[54] Zhang P., Yang G., Li F., Shi J., Zhong H. (2022). Direct in Situ Photolithography of Perovskite Quantum Dots Based on Photocatalysis of Lead Bromide Complexes. Nat. Commun..

[55] Wu C.Y., Hsieh H., Lee Y.C. (2019). Contact Photolithography at Sub-Micrometer Scale Using a Soft Photomask. Micromachines.

[56] Seisyan R.P. (2011). Nanolithography in Microelectronics: A Review. Tech. Phys..

[57] Pugachev M.V., Duleba A.I., Galiullin A.A., Kuntsevich A.Y. (2021). Micromask Lithography for Cheap and Fast 2D Materials Microstructures Fabrication. Micromachines.

[58] Cerrina F., Grenci G. (2016). X-ray Lithography. Reference Module in Materials Science and Materials Engineering.

[59] Jain N.K., Chaubey S.K., Hashmi M.S.J. (2017). 1.17 Review of Miniature Gear Manufacturing: Comprehensive Materials Finishing.

[60] Nguyen N.-T., Nguyen N.T. (2012). Fabrication Technologies. Micro and Nano Technologies, Micromixers.

[61] Gentselev A.N., Baev S.G. (2022). Production of Planar Elements of Terahertz Optics by Means of Deep X-ray Lithography. Optoelectron. Instrum. Data Process..

[62] Lohse M., Heinrich M., Grützner S., Haase A., Ramos I., Salado C., Thesen M.W., Grützner G. (2021). Versatile Fabrication Method for Multiscale Hierarchical Structured Polymer Masters Using a Combination of Photo- and Nanoimprint Lithography. Micro Nano Eng..

[63] Choi J., Lee C.C., Park S. (2019). Scalable Fabrication of Sub-10 nm Polymer Nanopores for DNA Analysis. Microsyst. Nanoeng..

[64] Sahin O., Ashokkumar M., Ajayan P.M., Balakrishnan P., Sreekala M.S., Thomas S. (2018). 3—Micro- and nanopatterning of biomaterial surfaces. Woodhead Publishing Series in Biomaterials, Fundamental Biomaterials: Metals.

[65] Kane R.S., Takayama S., Ostuni E., Ingber D.E., Whitesides G.M. (1999). Patterning Proteins and Cells Using Soft Lithography. Biomaterials.

[66] Abdelgawad M., Watson M.W.L., Young E.W.K., Mudrik J.M., Ungrin M.D., Wheeler A.R. (2008). Soft Lithography: Masters on Demand. Lab Chip.

[67] Lipomi D.J., Martinez R.V., Cademartiri L., Whitesides G.M. (2012). Soft Lithographic Approaches to Nanofabrication.

[68] Khademhosseini A., Du Y., Rajalingam B., Vacanti J.P., Langer R.S., Polak J., Mantalaris S., Harding S.E. (2008). Microscale Technologies for Tissue Engineering. Advances in Tissue Engineering.

[69] Gan Z., Najafidehaghani E., Han S.H., Shradha S., Abtahi F., Neumann C., Picker J., Vogl T., Hübner U., Eilenberger F. (2022). Patterned Growth of Transition Metal Dichalcogenide Monolayers and Multilayers for Electronic and Optoelectronic Device Applications. Small Methods.

[70] Garcia R., Knoll A.W., Riedo E. (2014). Advanced Scanning Probe Lithography. Nat. Nanotechnol..

[71] Fischer A.C., Mäntysalo M., Niklaus F. (2020). Inkjet Printing, Laser-Based Micromachining, and Micro–3D Printing Technologies for MEMS. Handbook of Silicon Based MEMS Materials and Technologies.

[72] Bruchhaus L., Mazarov P., Bischoff L., Gierak J., Wieck A.D., Hövel H. (2017). Comparison of Technologies for Nano Device Prototyping with a Special Focus on Ion Beams: A Review. Appl. Phys. Rev..

[73] Economou N., Ward B., Morgan J., Notte J. (2007). Helium Ion Microscopy: An Introduction. Innov. Pharm. Technol..

[74] Huth M., Porrati F., Dobrovolskiy O.V. (2018). Focused Electron Beam Induced Deposition Meets Materials Science. Microelectron. Eng..

[75] Ven Kouwen L., Botman A., Hagen C.W. (2009). Focused Electron-Beam-Induced Deposition of 3 Nm Dots in a Scanning Electron Microscope. Nano Lett..

[76] Fürjes P. (2019). Controlled Focused Ion Beam Milling of Composite Solid State Nanopore Arrays for Molecule Sensing. Micromachines.

[77] Martinez-Chapa S.O., Salazar A., Madou M.J. (2019). Two-Photon Polymerization as a Component of Desktop-Integrated Manufacturing Platforms.

[78] Charlton M.D.B. (2013). Photonic Crystal Nitride LEDs.

[79] Tu M., Xia B., Kravchenko D.E., Tietze M.L., Cruz A.J., Stassen I., Hauffman T., Teyssandier J., De Feyter S., Wang Z. (2021). Direct X-ray and Electron-Beam Lithography of Halogenated Zeolitic Imidazolate Frameworks. Nat. Mater..

[80] Bokov D., Turki Jalil A., Chupradit S., Suksatan W., Javed Ansari M., Shewael I.H., Valiev G.H., Kianfar E. (2021). Nanomaterial by Sol-Gel Method: Synthesis and Application. Adv. Mater. Sci. Eng..

[81] Yilmaz E., Soylak M. (2020). Functionalized Nanomaterials for Sample Preparation Methods. Handbook of Nanomaterials in Analytical Chemistry.

[82] Pajonk G.M. (1989). Drying methods preserving the textural Properties of gels. J. Phys. Colloques 50..

[83] Yu H.D., Regulacio M.D., Ye E., Han M.Y. (2013). Chemical Routes to Top-down Nanofabrication. Chem. Soc. Rev..

[84] Sun L., Yuan G., Gao L., Yang J., Chhowalla M., Gharahcheshmeh M.H., Gleason K.K., Choi Y.S., Hong B.H., Liu Z. (2021). Chemical Vapour Deposition. Nat. Rev. Methods Prim..

[85] Oviroh P.O., Akbarzadeh R., Pan D., Coetzee R.A.M., Jen T.C. (2019). New Development of Atomic Layer Deposition: Processes, Methods and Applications. Sci. Technol. Adv. Mater..

[86] Leskelä M., Niinistö J., Ritala M. (2014). Atomic Layer Deposition. Comprehensive Materials Processing.

[87] Xiong S., Qian X., Zhong Z., Wang Y. (2022). Atomic Layer Deposition for Membrane Modification, Functionalization and Preparation: A Review. J. Memb. Sci..

[88] Nur O., Willander M. (2020). Conventional Nanofabrication Methods. Low Temperature Chemical Nanofabrication.

[89] Wang S., Gao L., Thomas S., Grohens Y., Pottathara Y.B. (2019). Laser-Driven Nanomaterials and Laser-Enabled Nanofabrication for Industrial Applications. Micro and Nano Technologies, Industrial Applications of Nanomaterials.

[90] Cho A.Y., Arthur J.R. (1975). Molecular Beam Epitaxy. Prog. Solid State Chem..

[91] Adachi H., Wasa K. (2012). Thin Films and Nanomaterials. Handbook of Sputtering Technology.

[92] Lombardo D., Kiselev M.A., Magazù S., Calandra P. (2015). Amphiphiles Self-Assembly: Basic Concepts and Future Perspectives of Supramolecular Approaches. Adv. Condens. Matter Phys..

[93] Rahmawan Y., Xu L., Yang S. (2013). Self-Assembly of Nanostructures towards Transparent, Superhydrophobic Surfaces. J. Mater. Chem. A.

[94] Mijatovic D., Eijkel J.C.T., Van Den Berg A. (2005). Technologies for Nanofluidic Systems: Top-down vs. Bottom-up—A Review. Lab Chip.

[95] Mourdikoudis S., Pallares R.M., Thanh N.T.K. (2018). Characterization Techniques for Nanoparticles: Comparison and Complementarity upon Studying Nanoparticle Properties. Nanoscale.

[96] Birkholz M. (2006). Thin Film Analysis by X-ray Scattering.

[97] Yano J., Yachandra V.K. (2009). X-ray Absorption Spectroscopy. Photosynth. Res..

[98] Singh A.K. (2016). Experimental Methodologies for the Characterization of Nanoparticles. Engineered Nanoparticles.

[99] Engelhard M.H., Droubay T.C., Du Y. (2017). X-ray Photoelectron Spectroscopy Applications. Encyclopedia of Spectroscopy and Spectrometry.

[100] Janssens M. (2006). Fundamental Measurement Techniques. Flammability Testing of Materials Used in Construction, Transport and Mining.

[101] Koutcher J.A., Burt C.T. (1984). Principles of Nuclear Magnetic Resonance. J. Nucl. Med..

[102] Nasrollahzadeh M., Atarod M., Sajjadi M., Sajadi S.M., Issaabadi Z. (2019). Plant-Mediated Green Synthesis of Nanostructures: Mechanisms, Characterization, and Applications. Interface Sci. Technol..

[103] Bottom R. (2008). Thermogravimetric Analysis. Principles and Applications of Thermal Analysis.

[104] Venkatachalam S. (2016). Ultraviolet and Visible Spectroscopy Studies of Nanofillers and Their Polymer Nanocomposites. Spectroscopy of Polymer Nanocomposites.

[105] Stetefeld J., McKenna S.A., Patel T.R. (2016). Dynamic Light Scattering: A Practical Guide and Applications in Biomedical Sciences. Biophys. Rev..

[106] Minelli C., Sikora A., Garcia-Diez R., Sparnacci K., Gollwitzer C., Krumrey M., Shard A.G. (2018). Measuring the Size and Density of Nanoparticles by Centrifugal Sedimentation and Flotation. Anal. Methods.

[107] Goenaga-Infante H., Bartczak D. (2020). Single Particle Inductively Coupled Plasma Mass Spectrometry (SpICP-MS). Characterization of Nanoparticles.

[108] Zhao J., Liu X. (2022). Electron Microscopic Methods (TEM, SEM and Energy Dispersal Spectroscopy). Reference Module in Earth Systems and Environmental Sciences.

[109] Vlasov I.I., Turner S., Van Tendeloo G., Shiryaev A.A. (2012). Recent Results on Characterization of Detonation Nanodiamonds. Ultananocrystalline Diamond.

[110] Mehrpouya M., Lavvafi H., Darafsheh A. (2018). Microstructural Characterization and Mechanical Reliability of Laser-Machined Structures. Advances in Laser Materials Processing.

[111] Raval N., Maheshwari R., Kalyane D., Youngren-Ortiz S.R., Chougule M.B., Tekade R.K. (2019). Importance of Physicochemical Characterization of Nanoparticles in Pharmaceutical Product Development. Basic Fundamentals of Drug Delivery.

[112] Vahabi S., Nazemi Salman B., Javanmard A. (2013). Atomic Force Microscopy Application in Biological Research: A Review Study. Iran. J. Med. Sci..

[113] Ilyin A.M. (2017). Auger Electron Spectroscopy. Microscopy Methods in Nanomaterials Characterization.

[114] Rades S., Wirth T., Unger W. (2014). Investigation of Silica Nanoparticles by Auger Electron Spectroscopy (AES). Surf. Interface Anal..

[115] Xu M., Fujita D., Gao J., Hanagata N. (2010). Auger Electron Spectroscopy: A Rational Method for Determining Thickness of Graphene Films. ACS Nano.

[116] Vladitsi M., Nikolaou C., Kalogiouri N.P., Samanidou V.F. (2022). Analytical Methods for Nanomaterial Determination in Biological Matrices. Methods Protoc..

[117] Brandt A., Kees K., Leopold K. (2020). Characterization of Various Metal Nanoparticles by Graphite Furnace Atomic Absorption Spectrometry: Possibilities and Limitations with Regard to Size and Shape. J. Anal. At. Spectrom..

[118] Darienzo R.E., Wang J., Chen O., Sullivan M., Mironava T., Kim H., Tannenbaum R. (2019). Surface-Enhanced Raman Spectroscopy Characterization of Breast Cell Phenotypes: Effect of Nanoparticle Geometry. ACS Appl. Nano Mater..

[119] Monshi A., Foroughi M.R., Monshi M.R. (2012). Modified Scherrer Equation to Estimate More Accurately Nano-Crystallite Size Using XRD. World J. Nano Sci. Eng..

[120] Velavan S., Amargeetha A. (2018). X-ray Diffraction (XRD) and Energy Dispersive Spectroscopy (EDS) Analysis of Silver Nanoparticles Synthesized from Erythrina Indica Flowers. Nanosci. Technol. Open Access.

[121] De Groot F. (2001). High-Resolution X-ray Emission and X-ray Absorption Spectroscopy. Chem. Rev..

[122] Hummer A.A., Rompel A. (2013). X-ray Absorption Spectroscopy: A Tool to Investigate the Local Structure of Metal-Based Anticancer Compounds In Vivo.

[123] Lopes C.W., Cerrillo J.L., Palomares A.E., Rey F., Agostini G. (2018). An in Situ XAS Study of the Activation of Precursor-Dependent Pd Nanoparticles. Phys. Chem. Chem. Phys..

[124] Li T., Senesi A.J., Lee B. (2016). Small Angle X-ray Scattering for Nanoparticle Research. Chem. Rev..

[125] Ren Y., Ma Z., Bruce P.G. (2012). Ordered Mesoporous Metal Oxides: Synthesis and Applications. Chem. Soc. Rev..

[126] Garcia P.R.A.F., Prymak O., Grasmik V., Pappert K., Wlysses W., Otubo L., Epple M., Oliveira C.L.P. (2020). An in Situ SAXS Investigation of the Formation of Silver Nanoparticles and Bimetallic Silver–Gold Nanoparticles in Controlled Wet-Chemical Reduction Synthesis. Nanoscale Adv..

[127] Oswald S. (2013). X-ray Photoelectron Spectroscopy in Analysis of Surfaces.

[128] Baer D.R. (2020). Guide to Making XPS Measurements on Nanoparticles. J. Vac. Sci. Technol. A.

[129] Korin E., Froumin N., Cohen S. (2017). Surface Analysis of Nanocomplexes by X-ray Photoelectron Spectroscopy (XPS). ACS Biomater. Sci. Eng..

[130] Sublemontier O., Nicolas C., Aureau D., Patanen M., Kintz H., Liu X., Gaveau M.-A., Le Garrec J.-L., Robert E., Barreda F.-A. (2014). X-ray Photoelectron Spectroscopy of Isolated Nanoparticles. J. Phys. Chem. Lett..

[131] Pirozzi N.M., Kuipers J., Giepmans B.N.G. (2021). Sample Preparation for Energy Dispersive X-ray Imaging of Biological Tissues.

[132] Girão A.V., Caputo G., Ferro M.C. (2017). Application of Scanning Electron Microscopy–Energy Dispersive X-ray Spectroscopy (SEM-EDS). Compr. Anal. Chem..

[133] Scimeca M., Bischetti S., Lamsira H.K., Bonfiglio R., Bonanno E. (2018). Energy Dispersive X-ray (EDX) Microanalysis: A Powerful Tool in Biomedical Research and Diagnosis. Eur. J. Histochem..

[134] Hamuyuni J., Daramola M.O., Oluwasina O.O., Wang U.W., Juaristi E. (2017). Energy-Dispersive X-ray Spectroscopy: Theory and Application in Engineering and Science. Encyclopedia of Physical Organic Chemistry.

[135] Patri A., Umbreit T., Zheng J., Nagashima K., Goering P., Francke-Carroll S., Gordon E., Weaver J., Miller T., Sadrieh N. (2009). Energy Dispersive X-ray Analysis of Titanium Dioxide Nanoparticle Distribution after Intravenous and Subcutaneous Injection in Mice. J. Appl. Toxicol..

[136] Vladár A.E., Hodoroaba V.-D. (2020). Characterization of Nanoparticles by Scanning Electron Microscopy. Characterization of Nanoparticles.

[137] Vernon-Parry K.D. (2000). Scanning Electron Microscopy: An Introduction. III-Vs Rev..

[138] Suga M., Asahina S., Sakuda Y., Kazumori H., Nishiyama H., Nokuo T., Alfredsson V., Kjellman T., Stevens S.M., Cho H.S. (2014). Recent Progress in Scanning Electron Microscopy for the Characterization of Fine Structural Details of Nano Materials. Prog. Solid State Chem..

[139] Mohammed A., Abdullah A. Scanning Electron Microscopy (Sem): A Review. Proceedings of the 2018 International Conference on Hydraulics and Pneumatics—HERVEX.

[140] Zheng J., Nagashima K., Parmiter D., de la Cruz J., Patri A.K. (2011). SEM X-ray Microanalysis of Nanoparticles Present in Tissue or Cultured Cell Thin Sections. Methods Mol. Biol..

[141] Malatesta M. (2021). Transmission Electron Microscopy as a Powerful Tool to Investigate the Interaction of Nanoparticles with Subcellular Structures. Int. J. Mol. Sci..

[142] Tushar R.G., Babita A.R. (2013). Transmission Electron Microscopy-An Overview. Int. Res. J. Invent. Pharm. Sci..

[143] Cheng Z., Wang C., Wu X., Chu J. (2022). Review in Situ Transmission Electron Microscope with Machine Learning. J. Semicond..

[144] Franken L.E., Grünewald K., Boekema E.J., Stuart M.C.A. (2020). A Technical Introduction to Transmission Electron Microscopy for Soft-Matter: Imaging, Possibilities, Choices, and Technical Developments. Small.

[145] Walther T. (2017). Transmission Electron Microscopy of Nanostructures. Microscopy Methods in Nanomaterials Characterization.

[146] Ghosh S., Basu R.N. (2019). Nanoscale Characterization. Noble Metal-Metal Oxide Hybrid Nanoparticles.

[147] Aliofkhazraei M., Ali N. (2014). AFM Applications in Micro/Nanostructured Coatings. Comprehensive Materials Processing.

[148] Farré M., Barceló D. (2012). Introduction to the Analysis and Risk of Nanomaterials in Environmental and Food Samples. Compr. Anal. Chem..

[149] Sinha Ray S. (2013). Structure and Morphology Characterization Techniques. Clay-Containing Polymer Nanocomposites.

[150] Rao A., Schoenenberger M., Gnecco E., Glatzel T., Meyer E., Brändlin D., Scandella L. (2007). Characterization of Nanoparticles Using Atomic Force Microscopy. J. Phys. Conf. Ser..

[151] Groarke R., Vijayaraghavan R.K., Powell D., Rennie A., Brabazon D. (2021). Powder Characterization—Methods, Standards, and State of the Art. Fundamentals of Laser Powder Bed Fusion of Metals.

[152] Sindhu R., Binod P., Pandey A. (2015). Microbial Poly-3-Hydroxybutyrate and Related Copolymers. Industrial Biorefineries & White Biotechnology.

[153] Torres-Rivero K., Bastos-Arrieta J., Fiol N., Florido A. (2021). Metal and Metal Oxide Nanoparticles: An Integrated Perspective of the Green Synthesis Methods by Natural Products and Waste Valorization: Applications and Challenges. Compr. Anal. Chem..

[154] Devaraj P., Kumari P., Aarti C., Renganathan A. (2013). Synthesis and Characterization of Silver Nanoparticles Using Cannonball Leaves and Their Cytotoxic Activity against MCF-7 Cell Line. J. Nanotechnol..

[155] Plüisch C.S., Stuckert R., Wittemann A. (2021). Direct Measurement of Sedimentation Coefficient Distributions in Multimodal Nanoparticle Mixtures. Nanomaterials.

[156] Laborda F., Bolea E., Jimenez M.S. (2020). Inductively Coupled Plasma Mass Spectrometry for Nanomaterial Analysis. 21st Century Nanoscience—A Handbook.

[157] Fréchette-Viens L., Hadioui M., Wilkinson K.J. (2019). Quantification of ZnO Nanoparticles and Other Zn Containing Colloids in Natural Waters Using a High Sensitivity Single Particle ICP-MS. Talanta.

[158] Murphy K.E., Liu J., Montoro Bustos A.R., Johnson M.E., Winchester M.R. (2016). Characterization of Nanoparticle Suspensions Using Single Particle Inductively Coupled Plasma Mass Spectrometry. National Institute of Standards and Technology Special Publication 1200-21.

[159] Ren X., Zhang F., Guo B., Gao N., Zhang X. (2019). Synthesis of N-Doped Micropore Carbon Quantum Dots with High Quantum Yield and Dual-Wavelength Photoluminescence Emission from Biomass for Cellular Imaging. Nanomaterials.

[160] Sabri M., Habibi-Yangjeh A., Vadivel S. (2019). Activation of Persulfate Ions by TiO_2_/Carbon Dots Nanocomposite under Visible Light for Photocatalytic Degradations of Organic Contaminants. J. Mater. Sci. Mater. Electron..

[161] Mohan V.B., Jayaraman K., Bhattacharyya D. (2020). Brunauer–Emmett–Teller (BET) Specific Surface Area Analysis of Different Graphene Materials: A Comparison to Their Structural Regularity and Electrical Properties. Solid State Commun..

[162] Saadatkhah N., Carillo Garcia A., Ackermann S., Leclerc P., Latifi M., Samih S., Patience G.S., Chaouki J. (2020). Experimental Methods in Chemical Engineering: Thermogravimetric Analysis—TGA. Can. J. Chem. Eng..

[163] Vyazovkin S., Burnham A.K., Criado J.M., Pérez-Maqueda L.A., Popescu C., Sbirrazzuoli N. (2011). ICTAC Kinetics Committee Recommendations for Performing Kinetic Computations on Thermal Analysis Data. Thermochim. Acta.

[164] Li Y., Liu Z., Wu Y., Chen J., Zhao J., Jin F., Na P. (2018). Carbon Dots-TiO2 Nanosheets Composites for Photoreduction of Cr(VI) under Sunlight Illumination: Favorable Role of Carbon Dots. Appl. Catal. B Environ..

[165] Bannov A.G., Popov M.V., Kurmashov P.B. (2020). Thermal Analysis of Carbon Nanomaterials: Advantages and Problems of Interpretation. J. Therm. Anal. Calorim..

[166] Farivar F., Lay Yap P., Karunagaran R.U., Losic D. (2021). Thermogravimetric Analysis (TGA) of Graphene Materials: Effect of Particle Size of Graphene, Graphene Oxide and Graphite on Thermal Parameters. C.

[167] John B.K., Abraham T., Mathew B. (2022). A Review on Characterization Techniques for Carbon Quantum Dots and Their Applications in Agrochemical Residue Detection. J. Fluoresc..

[168] Jiang L., Gao L., Sun J. (2003). Production of Aqueous Colloidal Dispersions of Carbon Nanotubes. J. Colloid Interface Sci..

[169] Pu N.W., Wang C.A., Liu Y.M., Sung Y., Wang D.S., Ger M. (2012). Der Dispersion of Graphene in Aqueous Solutions with Different Types of Surfactants and the Production of Graphene Films by Spray or Drop Coating. J. Taiwan Inst. Chem. Eng..

[170] Rashmi W., Ismail A.F., Sopyan I., Jameel A.T., Yusof F., Khalid M., Mubarak N.M. (2011). Stability and Thermal Conductivity Enhancement of Carbon Nanotube Nanofluid Using Gum Arabic. J. Exp. Nanosci..

[171] Abdolkarimi-Mahabadi M., Bayat A., Mohammadi A. (2021). Use of UV-Vis Spectrophotometry for Characterization of Carbon Nanostructures: A Review. Theor. Exp. Chem..

[172] Liu J.C., Wang Y.J., Liu L., Sun D.D. (2011). High-Quality Reduced Graphene Oxide-Nanocrystalline Platinum Hybrid Materials Prepared by Simultaneous Co-Reduction of Graphene Oxide and Chloroplatinic Acid. Nanoscale Res. Lett..

[173] Wang G., Shen X., Yao J., Park J. (2009). Graphene Nanosheets for Enhanced Lithium Storage in Lithium Ion Batteries. Carbon N. Y..

[174] Wang G., Wang B., Park J., Yang J., Shen X., Yao J. (2009). Synthesis of Enhanced Hydrophilic and Hydrophobic Graphene Oxide Nanosheets by a Solvothermal Method. Carbon N. Y..

[175] Paredes J.I., Villar-Rodil S., Martínez-Alonso A., Tascón J.M.D. (2008). Graphene Oxide Dispersions in Organic Solvents. Langmuir.

[176] Georgakilas V., Otyepka M., Bourlinos A.B., Chandra V., Kim N., Kemp K.C., Hobza P., Zboril R., Kim K.S. (2012). Functionalization of Graphene: Covalent and Non-Covalent Approaches, Derivatives and Applications. Chem. Rev..

[177] Dager A., Uchida T., Maekawa T., Tachibana M. (2019). Synthesis and Characterization of Mono-Disperse Carbon Quantum Dots from Fennel Seeds: Photoluminescence Analysis Using Machine Learning. Sci. Rep..

[178] Reinert L., Zeiger M., Suárez S., Presser V., Mücklich F. (2015). Dispersion Analysis of Carbon Nanotubes, Carbon Onions, and Nanodiamonds for Their Application as Reinforcement Phase in Nickel Metal Matrix Composites. RSC Adv..

[179] Smith B., Wepasnick K., Schrote K.E., Bertele A.R., Ball W.P., O’Melia C., Fairbrother D.H. (2009). Colloidal Properties of Aqueous Suspensions of Acid-Treated, Multi-Walled Carbon Nanotubes. Environ. Sci. Technol..

[180] Xu H., Cheng X., Zhong J., Meng J., Yang M., Jia F., Xu Z., Kong H. (2011). Characterization of Multiwalled Carbon Nanotubes Dispersing in Water and Association with Biological Effects. J. Nanomater..

[181] Wang Y.M., Zhou D.M., Yuan X.Y., Zhang X.H., Li Y. (2018). Modeling the Interaction and Toxicity of Cu-Cd Mixture to Wheat Roots Affected by Humic Acids, in Terms of Cell Membrane Surface Characteristics. Chemosphere.

[182] Kumar P., Chakraborty S., Chinthapudi E., Basu S., Narayan B. (2023). Rice Husk-Derived Silica Nanoparticles Using Optimized Titrant Concentration for the One-Step Nanofluid Preparation. Sustainable Chemical, Mineral and Material Processing.

[183] Amaro-Gahete J., Benítez A., Otero R., Esquivel D., Jiménez-Sanchidrián C., Morales J., Caballero Á., Romero-Salguero F. (2019). A Comparative Study of Particle Size Distribution of Graphene Nanosheets Synthesized by an Ultrasound-Assisted Method. Nanomaterials.

[184] Filipe V., Hawe A., Jiskoot W. (2010). Critical Evaluation of Nanoparticle Tracking Analysis (NTA) by NanoSight for the Measurement of Nanoparticles and Protein Aggregates. Pharm. Res..

[185] Lin P.C., Lin S., Wang P.C., Sridhar R. (2014). Techniques for Physicochemical Characterization of Nanomaterials. Biotechnol. Adv..

[186] Edwards E.R., Antunes E.F., Botelho E.C., Baldan M.R., Corat E.J. (2011). Evaluation of Residual Iron in Carbon Nanotubes Purified by Acid Treatments. Appl. Surf. Sci..

[187] Pourjavid M.R., Arabieh M., Yousefi S.R., Akbari Sehat A. (2016). Interference Free and Fast Determination of Manganese(II), Iron(III) and Copper(II) Ions in Different Real Samples by Flame Atomic Absorption Spectroscopy after Column Graphene Oxide-Based Solid Phase Extraction. Microchem. J..

[188] Gu J., Zhang X., Pang A., Yang J. (2016). Facile Synthesis and Photoluminescence Characteristics of Blue-Emitting Nitrogen-Doped Graphene Quantum Dots. Nanotechnology.

[189] Okamoto A., Shinohara H. (2005). Control of Diameter Distribution of Single-Walled Carbon Nanotubes Using the Zeolite-CCVD Method at Atmospheric Pressure. Carbon.

[190] Autumn K., Liang Y.A., Hsieh S.T., Zesch W., Chan W.P., Kenny T.W., Fearing R., Full R.J. (2000). Adhesive Force of a Single Gecko Foot-Hair. Nature.

[191] Gorb S.N., Varenberg M. (2007). Mushroom-Shaped Geometry of Contact Elements in Biological Adhesive Systems. J. Adhes. Sci. Technol..

[192] Gorb S., Varenberg M., Peressadko A., Tuma J. (2007). Biomimetic Mushroom-Shaped Fibrillar Adhesive Microstructure. J. R. Soc. Interface.

[193] Carbone G., Pierro E., Gorb S.N. (2011). Origin of the Superior Adhesive Performance of Mushroom-Shaped Microstructured Surfaces. Soft Matter.

[194] Shao J., Ding Y., Wang W., Mei X., Zhai H., Tian H., Li X., Liu B. (2014). Generation of Fully-Covering Hierarchical Micro-/Nano- Structures by Nanoimprinting and Modified Laser Swelling. Small.

[195] Paras, Kumar A. (2021). Smart Bioinspired Anti-Wetted Surfaces: Perspectives, Fabrication, Stability and Applications. Curr. Res. Green Sustain. Chem..

[196] Shateri-Khalilabad M., Yazdanshenas M.E., Etemadifar A. (2017). Fabricating Multifunctional Silver Nanoparticles-Coated Cotton Fabric. Arab. J. Chem..

[197] Bindra H.S., Krishna J., Kumeria T., Nayak R. Rapid and Facile Fabrication of Wafer Scale Silicon Hierarchical Structures with Broadband Ultra High Anti-Reflection Property. Proceedings of the 3rd International Electronic Conference on Materials Sciences.

[198] Cho S.J., Seok S.Y., Kim J.Y., Lim G., Lim H. (2013). One-Step Fabrication of Hierarchically Structured Silicon Surfaces and Modification of Their Morphologies Using Sacrificial Layers. J. Nanomater..

[199] Wang L., Li J., Song S., Li D., Fan C. (2009). Biomolecular Sensing via Coupling DNA-Based Recognition with Gold Nanoparticles. J. Phys. D. Appl. Phys..

[200] Jain P.K., Huang X., El-Sayed I.H., El-Sayed M.A. (2008). Noble Metals on the Nanoscale: Optical and Photothermal Properties and Some Applications in Imaging, Sensing, Biology, and Medicine. Acc. Chem. Res..

[201] Wei H., Wang E. (2013). Nanomaterials with Enzyme-like Characteristics (Nanozymes): Next-Generation Artificial Enzymes. Chem. Soc. Rev..

[202] Pavlov V., Xiao Y., Shlyahovsky B., Willner I. (2004). Aptamer-Functionalized Au Nanoparticles for the Amplified Optical Detection of Thrombin. J. Am. Chem. Soc..

[203] Leggett R., Lee-Smith E.E., Jickells S.M., Russell D.A. (2007). “Intelligent” Fingerprinting: Simultaneous Identification of Drug Metabolites and Individuals by Using Antibody-Functionalized Nanoparticles. Angew. Chemie.

[204] Hawkins M.J., Soon-Shiong P., Desai N. (2008). Protein Nanoparticles as Drug Carriers in Clinical Medicine. Adv. Drug Deliv. Rev..

[205] Ryan S., Kell A.J., van Faassen H., Tay L.-L., Simard B., MacKenzie R., Gilbert M., Tanha J. (2009). Single-Domain Antibody-Nanoparticles: Promising Architectures for Increased Staphylococcus Aureus Detection Specificity and Sensitivity. Bioconjug. Chem..

[206] Jeevanandam J., Barhoum A., Chan Y.S., Dufresne A., Danquah M.K. (2018). Review on Nanoparticles and Nanostructured Materials: History, Sources, Toxicity and Regulations. Beilstein J. Nanotechnol..

[207] Pandey S., Sharma K.H., Sharma A.K., Nerthigan Y., Hang D.-R., Wu H.-F. (2018). Comparative photothermal performance among various sub-stoichiometric 2D oxygen-deficient molybdenum oxide nanoflakes and in vivo toxicity. Chem. Eur. J..

[208] Bhaisare M.L., Sharma K.H., Lee J.-Y., Hang D.-R., Wu H.-F. (2016). Synthesis and characterization of two-dimensional carbon dots decorated with molybdenum oxide nanoflakes with various phases. New J. Chem..

[209] Nerthigan Y., Sharma A.K., Pandey S., Sharma K.H., Khan M.S., Hang D.-R., Wu H.-F. (2018). Glucose oxidase assisted visual detection of glucose using oxygen deficient α-MoO_3-x_ nanoflakes. Microchim. Acta.

[210] Sharma A.K., Pandey S., Sharma K.H., Nerthigan Y., Khan M.S., Hang D.-R., Wu H.-F. (2018). Two dimensional α-MoO_3-x_ nanoflakes as bare eye probe for hydrogen peroxide in biological fluids. Anal. Chim. Acta.

[211] Pandey S., Sharma A.K., Sharma K.H., Nerthigan Y., Khan M.S., Hang D.-R., Wu H.-F. (2018). Rapid naked eye detection of alkaline phosphatase using α-MoO_3-x_ nano-flakes. Sens. Actuators B Chem..

[212] Brueck S.R. (2004). Photonics in Nanotechnology. Conference on Lasers and Electro-Optics/International Quantum Electronics Conference and Photonic Applications Systems Technologies.

[213] Hang D.-R., Islam S.E., Sharma K.H., Chen C., Liang C.-T., Chou M.M.C. (2014). Optical characteristics of nonpolar *a*-plane ZnO thin film on (010) LiGaO_2_ substrate. Semicond. Sci. Technol..

[214] Hang D.R., Liang C.-T., Huang C.F., Chang Y.H., Chen Y.F., Jiang H.X., Lin J.Y. (2001). Effective mass of two-dimensional electron gas in an Al_0.2_Ga_0.8_N/GaN heterojunction. Appl. Phys. Lett..

[215] Hang D.-R., Chen Y.F., Jiang H.X., Lin J.Y. (2001). Al_x_Ga_1-x_N/GaN band offsets determined by deep-level emission. J. Appl. Phys..

[216] Hang D.-R., Huang C.F., Chen Y.F. (2006). Two-subband-populated AlGaN/GaN heterostructures probed by electrically detected and microwave-modulated magnetotransport measurements. Appl. Phys. Lett..

[217] Chou M.M.C., Chen C., Hang D.-R., Yang W.-T. (2011). Growth of nonpolar *m*-plane GaN epitaxial film on a lattice-matched (100) β-LiGaO_2_ substrate by chemical vapor deposition. Thin Solid Films.

[218] Chou M.M.C., Hang D.-R., Kalisch H., Jansen R.H., Dikme Y., Heuken M., Yablonskii G.P. (2007). Crystal growth and properties of LiAlO_2_ and nonpolar GaN on LiAlO_2_ substrate. J. Appl. Phys..

[219] Hang D.-R., Chou M.M.C., Chang L., Dikme Y., Heuken M. (2009). Growth and characterization of *m*-plane GaN-based layers on LiAlO_2_ (1 0 0) grown by MOVPE. J. Crystal Growth.

[220] Hang D.-R., Chou M.M.C., Chang L., Lin J.L., Heuken M. (2009). Optical characteristics of *m*-plane InGaN/GaN multiple quantum well grown on LiAlO_2_ (1 0 0) by MOVPE. J. Crystal Growth.

[221] Hang D.-R., Chou M.M.C., Mauder C., Heuken M. (2010). MOVPE growth and properties of non-polar InGaN/GaN multiple quantum wells on γ-LiAlO_2_ substrates. J. Crystal Growth.

[222] Hang D.-R., Islam S.E., Sharma K.H., Kuo S.-W., Zhang C.-Z., Wang J.-J. (2014). Annealing effects on the optical and morphological properties of ZnO nanorods on AZO substrate by using aqueous solution method at low temperature. Nanoscale Res. Lett..

[223] Hang D.-R., Islam S.E., Chen C.-H., Sharma K.H. (2016). Full solution-processed synthesis and mechanisms of a recyclable and bifunctional Au/ZnO plasmonic platform for enhanced UV/Vis photocatalysis and optical properties. Chem. Eur. J..

[224] Chen W.-J., Wu J.-K., Lin J.-C., Lo S.-T., Lin H.-D., Hang D.-R., Shin M.-F., Liang C.-T., Chang Y.H. (2013). Room-temperature violet luminescence and ultraviolet photodetection of Sb-doped ZnO/Al-doped ZnO homojunction array. Nanoscale Res. Lett..

[225] Wu J.-K., Chen W.-J., Chang Y.H., Chen Y.F., Hang D.-R., Liang C.-T., Lu J.-Y. (2013). Fabrication and photo-response of ZnO nanowiress/CuO coaxial heterojunction. Nanoscale Res. Lett..

[226] Chou M.M.C., Hang D.-R., Wang S.C., Chen C., Lee C.-Y. (2010). Growth and characterizations of nonpolar [1 1 −2 0] ZnO on [1 0 0] (La,Sr)(Al,Ta)O_3_ substrate by chemical vapor deposition. J. Crystal Growth.

[227] Hang D.-R., Sharma K.H., Islam S.E., Chen C., Chou M.M.C. (2014). Resonant Raman scattering and photoluminescent properties of nonpolar *a*-plane ZnO thin film on LiGaO_2_ substrate. Appl. Phys. Express.

[228] Nguyen H.P.T., Arafin S., Piao J., Cuong T.V. (2016). Nanostructured Optoelectronics: Materials and Devices. J. Nanomater..

[229] Aamir Iqbal M., Ashraf N., Shahid W., Awais M., Khan Durrani A., Shahzad K., Ikram M. (2021). Nanophotonics: Fundamentals, Challenges, Future Prospects and Applied Applications. Nonlinear Optics—Nonlinear Nanophotonics and Novel Materials for Nonlinear Optics.

[230] Ye Y., Wong Z.J., Lu X., Ni X., Zhu H., Chen X., Wang Y., Zhang X. (2015). Monolayer Excitonic Laser. Nat. Photonics.

[231] Meng Z., Stolz R.M., Mendecki L., Mirica K.A. (2019). Electrically-Transduced Chemical Sensors Based on Two-Dimensional Nanomaterials. Chem. Rev..

[232] Steinhubl S.R., Muse E.D., Topol E.J. (2015). The Emerging Field of Mobile Health. Sci. Transl. Med..

[233] Mu B., Zhang J., McNicholas T.P., Reuel N.F., Kruss S., Strano M.S. (2014). Recent Advances in Molecular Recognition Based on Nanoengineered Platforms. Acc. Chem. Res..

[234] Masuda Y. (2022). Recent advances in SnO_2_ nanostructure based gas sensors. Sens. Actuators B Chem..

[235] Jiahua F., Qiulin T., Wenyi L. (2016). Preparation of CNT-SnO_2_ Composites and Study on Formaldehyde Gas Sensitivity. Sci. Technol. Eng..

[236] Liu B., Zhou K. (2019). Recent Progress on Graphene-Analogous 2D Nanomaterials: Properties, Modeling and Applications. Prog. Mater. Sci..

[237] Wen W., Song Y., Yan X., Zhu C., Du D., Wang S., Asiri A.M., Lin Y. (2018). Recent Advances in Emerging 2D Nanomaterials for Biosensing and Bioimaging Applications. Mater. Today.

[238] Hang D.-R., Pan Y.-Q., Sharma K.H., Chou M.M.C., Islam S.E., Wu H.-F., Liang C.-T. (2020). 2D CTAB-MoSe_2_ nanosheets and 0D MoSe_2_ quantum dots: Facile top-down preparations and their peroxidase-like catalytic activity for colorimetric detection of hydrogen peroxide. Nanomaterials.

[239] Hang D.-R., Sun D.-Y., Chen C.-H., Wu H.-F., Chou M.M.C., Islam S.E., Sharma K.H. (2019). Facile bottom-up preparation of WS_2_-based water-soluble quantum dots as luminescent probes for hydrogen peroxide and glucose. Nanoscale Res. Lett..

[240] Avasare V., Zhang Z., Avasare D., Khan I., Qurashi A. (2015). Room-Temperature Synthesis of TiO_2_ Nanospheres and Their Solar Driven Photoelectrochemical Hydrogen Production. Int. J. Energy Res..

[241] Mueller N.C., Nowack B. (2008). Exposure Modeling of Engineered Nanoparticles in the Environment. Environ. Sci. Technol..

[242] Zhou Y., Dong C.-K., Han L., Yang J., Du X.-W. (2016). Top-Down Preparation of Active Cobalt Oxide Catalyst. ACS Catal..

[243] Wang D.-W., Su D. (2014). Heterogeneous Nanocarbon Materials for Oxygen Reduction Reaction. Energy Environ. Sci..

[244] Maarisetty D., Reeba M., Hang D.-R., Mohapatra P., Baral S.S. (2022). The role of material defects in the photocatalytic CO_2_ reduction: Interfacial properties, thermodynamics, kinetics and mechanism. J. CO2 Util..

[245] Li D., Baydoun H., Verani C.N., Brock S.L. (2016). Efficient Water Oxidation Using CoMnP Nanoparticles. J. Am. Chem. Soc..

[246] Fang X.-Q., Liu J.-X., Gupta V. (2013). Fundamental Formulations and Recent Achievements in Piezoelectric Nano-Structures: A Review. Nanoscale.

[247] Lei Y.-M., Huang W.-X., Zhao M., Chai Y.-Q., Yuan R., Zhuo Y. (2015). Electrochemiluminescence Resonance Energy Transfer System: Mechanism and Application in Ratiometric Aptasensor for Lead Ion. Anal. Chem..

[248] Gawande M.B., Goswami A., Felpin F.-X., Asefa T., Huang X., Silva R., Zou X., Zboril R., Varma R.S. (2016). Cu and Cu-Based Nanoparticles: Synthesis and Applications in Catalysis. Chem. Rev..

[249] Greeley J., Markovic N.M. (2012). The Road from Animal Electricity to Green Energy: Combining Experiment and Theory in Electrocatalysis. Energy Environ. Sci..

[250] Liu J., Liu Y., Liu N., Han Y., Zhang X., Huang H., Lifshitz Y., Lee S.-T., Zhong J., Kang Z. (2015). Metal-Free Efficient Photocatalyst for Stable Visible Water Splitting via a Two-Electron Pathway. Science.

[251] Maarisetty D., Hang D.-R., Chou M.M.C., Parida S. (2022). Tuning the Ni/Co ratios and surface concentration of reduced molybdenum states for enhanced electrocatalytic performance in trimetallic molybdates: OER, HER, and MOR activity. ACS Appl. Energy Mater..

[252] Hang D.-R., Sharma K.H., Chen C.-H., Islam S.E. (2016). Enhanced photocatalytic performance of ZnO nanorods coupled by two-dimensional α-MoO_3_ nanoflakes under UV and visible light irradiation. Chem. Eur. J..

[253] Islam S.E., Hang D.-R., Chen C.-H., Sharma K.H. (2018). Facile and cost-efficient synthesis of quasi-0D/2D ZnO/MoS_2_ nanocomposites for highly enhanced visible-light-driven photocatalytic degradation of organic pollutants and antibiotics. Chem. Eur. J..

[254] Islam S.E., Hang D.-R., Chen C.-H., Chou M.M.C., Liang C.-T., Sharma K.H. (2021). Rational design of hetero-dimensional C-ZnO/MoS_2_ nanocomposite anchored on 3D mesoporous carbon framework towards synergistically enhanced stability and efficient visible-light-driven photocatalytic activity. Chemosphere.

[255] Sharma K.H., Hang D.-R., Bolloju S., Lee J.-T., Wu H.-F., Islam S.E., Chou M.M.C., Liang C.-T., Srivastava R.R. (2021). Two-dimensional molybdenum trioxide nanoflakes wrapped with interlayer-expanded molybdenum disulfide nanosheets: Superior performances in supercapacitive energy storage and visible-light-driven photocatalysis. Int. J. Hydrog. Energy.

[256] Ning F., Shao M., Xu S., Fu Y., Zhang R., Wei M., Evans D.G., Duan X. (2016). TiO_2_/Graphene/NiFe-Layered Double Hydroxide Nanorod Array Photoanodes for Efficient Photoelectrochemical Water Splitting. Energy Environ. Sci..

[257] Ulaeto S.B., Pancrecious J.K., Rajan T.P.D., Pai B.C. (2019). Smart Coatings. Noble Metal-Metal Oxide Hybrid Nanoparticles.

[258] Zou L., Lan C., Li X., Zhang S., Qiu Y., Ma Y. (2015). Superhydrophobization of Cotton Fabric with Multiwalled Carbon Nanotubes for Durable Electromagnetic Interference Shielding. Fibers Polym..

[259] Aminayi P., Abidi N. (2015). Ultra-Oleophobic Cotton Fabric Prepared Using Molecular and Nanoparticle Vapor Deposition Methods. Surf. Coatings Technol..

[260] Koch K., Bhushan B., Jung Y.C., Barthlott W. (2009). Fabrication of Artificial Lotus Leaves and Significance of Hierarchical Structure for Superhydrophobicity and Low Adhesion. Soft Matter.

[261] Jung Y.C., Bhushan B. (2009). Mechanically Durable Carbon Nanotube−Composite Hierarchical Structures with Superhydrophobicity, Self-Cleaning, and Low-Drag. ACS Nano.

[262] Bravo J., Zhai L., Wu Z., Cohen R.E., Rubner M.F. (2007). Transparent Superhydrophobic Films Based on Silica Nanoparticles. Langmuir.

[263] Ubaid F., Radwan A.B., Naeem N., Shakoor R.A., Ahmad Z., Montemor M.F., Kahraman R., Abdullah A.M., Soliman A. (2019). Multifunctional Self-Healing Polymeric Nanocomposite Coatings for Corrosion Inhibition of Steel. Surf. Coatings Technol..

[264] Birkett M., Dover L., Cherian Lukose C., Wasy Zia A., Tambuwala M.M., Serrano-Aroca Á. (2022). Recent Advances in Metal-Based Antimicrobial Coatings for High-Touch Surfaces. Int. J. Mol. Sci..

[265] Salesa B., Assis M., Andrés J., Serrano-Aroca Á. (2021). Carbon Nanofibers versus Silver Nanoparticles: Time-Dependent Cytotoxicity, Proliferation, and Gene Expression. Biomedicines.

[266] Zhao J., Cai X.M., Tang H.Q., Liu T., Gu H.Q., Cui R.Z. (2009). Bactericidal and Biocompatible Properties of TiN/Ag Multilayered Films by Ion Beam Assisted Deposition. J. Mater. Sci. Mater. Med..

[267] Mejía H.D., Echavarría A.M., Bejarano G.G. (2019). Influence of Ag-Cu Nanoparticles on the Microstructural and Bactericidal Properties of TiAlN(Ag,Cu) Coatings for Medical Applications Deposited by Direct Current (DC) Magnetron Sputtering. Thin Solid Films.

[268] Esteban-Tejeda L., Prado C., Cabal B., Sanz J., Torrecillas R., Moya J.S. (2015). Antibacterial and Antifungal Activity of ZnO Containing Glasses. PLoS ONE.

[269] Hizal F., Rungraeng N., Lee J., Jun S., Busscher H.J., van der Mei H.C., Choi C.-H. (2017). Nanoengineered Superhydrophobic Surfaces of Aluminum with Extremely Low Bacterial Adhesivity. ACS Appl. Mater. Interfaces.

[270] Bartlet K., Movafaghi S., Dasi L.P., Kota A.K., Popat K.C. (2018). Antibacterial Activity on Superhydrophobic Titania Nanotube Arrays. Colloids Surf. B Biointerfaces.

[271] Liu X., Li C., Lv J., Huang F., An Y., Shi L., Ma R. (2020). Glucose and H_2_O_2_ Dual-Responsive Polymeric Micelles for the Self-Regulated Release of Insulin. ACS Appl. Bio Mater..

[272] Zhou W., Yin Z., Du Y., Huang X., Zeng Z., Fan Z., Liu H., Wang J., Zhang H. (2013). Synthesis of Few-Layer MoS_2_ Nanosheet-Coated TiO_2_ Nanobelt Heterostructures for Enhanced Photocatalytic Activities. Small.

[273] Nasir J.A., Rehman Z.U., Shah S.N.A., Khan A., Butler I.S., Catlow C.R.A. (2020). Recent Developments and Perspectives in CdS-Based Photocatalysts for Water Splitting. J. Mater. Chem. A.

[274] Liu X., Li J., Yao W. (2020). CdS@MoS_2_ Hetero-structured Nanocomposites Are Highly Effective Photo-Catalysts for Organic Dye Degradation. ACS Omega.

[275] Xu X., Xiong F., Meng J., Wang X., Niu C., An Q., Mai L. (2020). Vanadium-Based Nanomaterials: A Promising Family for Emerging Metal-Ion Batteries. Adv. Funct. Mater..

[276] Zhang Q., Cheng X., Wang C., Rao A., Lu B. (2021). Sulfur-Assisted Large-Scale Synthesis of Graphene Microspheres for Superior Potassium-Ion Batteries. Energy Environ. Sci..

[277] Desalegn B.Z., Jadhav H.S., Seo J.G. (2019). Highly efficient g-C_3_N_4_ nanorods with dual active sites as an electrocatalyst for the oxygen evolution reaction. ChemCatChem.

